# Empagliflozin Alleviates Hepatic Steatosis by Activating the AMPK-TET2-Autophagy Pathway *in vivo* and *in vitro*

**DOI:** 10.3389/fphar.2020.622153

**Published:** 2021-01-20

**Authors:** Ting Li, Ting Fang, Linxin Xu, Xiangyang Liu, Xiaoyu Li, Mei Xue, Xiaochen Yu, Bei Sun, Liming Chen

**Affiliations:** NHC Key Laboratory of Hormones and Development, Tianjin Key Laboratory of Metabolic Diseases, Chu Hsien-I Memorial Hospital and Tianjin Institute of Endocrinology, Tianjin Medical University, Tianjin, China

**Keywords:** empagliflozin, autophagy, diabetes, metabolic associated fatty liver disease, lipid accumulation, ten-eleven translocation 2

## Abstract

**Background:** Metabolic associated fatty liver disease (MAFLD), characterized by hepatic lipid accumulation and fatty degeneration, is intertwined with obesity and type 2 diabetes mellitus (T2DM). Empagliflozin is a sodium-glucose cotransporter-2 inhibitor that effectively lowers blood glucose, but its effect on MAFLD and associated mechanisms are not fully understood.

**Methods:** Eight-week-old db/db mice, an *in vivo* model, were administered empagliflozin or saline intragastrically. A hepatocyte steatosis model was established by inducing HL7702 cells with high glucose and palmitic acid and then treated with or without empagliflozin. The autophagy inhibitor (3-methyladenine, 3-MA) and AMP-activated protein kinase (AMPK) activator (AICAR)/inhibitor (Compound C) were used to determine the involvement of AMPK and autophagy in the regulation of lipid accumulation by empagliflozin. Ten-eleven translocation 2 (TET2) knockdown was achieved by siRNA transfection. Hepatic steatosis was evaluated by Oil Red O staining and triglyceride quantification. Immunohistochemistry, immunofluorescence, and western blot were performed to assess protein levels.

**Results:** Empagliflozin alleviated liver steatosis in db/db mice and reduced triglyceride content and lipid accumulation in the hepatocyte steatosis model. Empagliflozin elevated autophagy, accompanied by an increase in *p*-AMPK and TET2. Both 3-MA and Compound C abolished the ability of empagliflozin to induce autophagy and reduce hepatic steatosis, while these effects could be recapitulated by AICAR treatment. TET2 knockdown resulted in autophagy inhibition and lipid accumulation despite empagliflozin treatment.

**Conclusion:** Empagliflozin improves hepatic steatosis through the AMPK-TET2-autophagy pathway. The use of empagliflozin as a treatment for preventing and treating MAFLD in patients with T2DM warrants further study.

## Introduction

Metabolic associated fatty liver disease (MAFLD), formerly named non-alcoholic fatty liver disease (NAFLD), inflicts a heavy health burden worldwide (Eslam et al., 2020). In absence of alcohol abuse, it encompasses a spectrum of liver disorders from simple steatosis to steatohepatitis, the latter of which can lead to progressive liver fibrosis (Lee et al., 2020), cirrhosis, and ultimately hepatocellular carcinoma (HCC) (Kanwal et al., 2019). MAFLD is epidemiologically related to obesity and type 2 diabetes mellitus (T2DM). Up to 70%–80% of patients with T2DM have MAFLD, and the concurrent conditions of MAFLD in T2DM are associated with more severe histological changes and worse outcomes than MAFLD alone (Targher et al., 2018). MAFLD arises from the imbalance between fat accumulation and β-oxidation and consequent excessive fat deposition in the liver. However, the exact molecular mechanisms underlying the pathogenesis and progression of MAFLD remain unclear, limiting the development of effective treatments for this disease. There is an urgent need to unravel the molecular pathogenesis of MAFLD and identify novel therapeutic targets.

Autophagy is a programmed degradation process that maintains cellular homeostasis under basal and stress conditions. It is a major pathway for lipid catabolism in hepatocytes. In this process, lipid droplets are enclosed by autophagosomes, which fuse with lysosomes and break down triglycerides into free fatty acids (Ueno and Komatsu, 2017). Dysfunction of autophagy has been observed in MAFLD (Fukuo et al., 2014), and autophagy deficient mice (*Atg*7^−/−^) exhibited increased hepatic fat accumulation and phenotypes that resembled the clinical presentations of MAFLD in humans (Singh et al., 2009). Also, autophagy was defective in ob/ob mice and in high-fat diet-induced obese mice, which showed substantial hepatic fatty infiltration; conversely, hepatic overexpression of *Atg7* prevented the development of fatty liver hepatic steatosis in these mice (Yang et al., 2010).

Ten-eleven translocation 2 (TET2) oxidizes 5-methycytosine (5-mC) to 5-hydroxymethylcytosine (5-hmC) and converts the latter to unmethylated cytosine by base excision repair and thymidine DNA glycosylase (Tahiliani et al., 2009). TET2-mediated demethylation is involved in various physical and pathological processes such as tumorigenesis (Pan et al., 2017), cell differentiation and proliferation (Wang et al., 2015b), and embryonic stem cell maintenance (von Meyenn et al., 2016). Emerging evidence has suggested a role of DNA demethylation in the pathogenesis of metabolic diseases (Volkmar et al., 2012; Multhaup et al., 2015). Upstream of TET2, AMP-activated protein kinase (AMPK) serves as a metabolic sensor and orchestrates epigenetic modifications by TET2 (Wu et al., 2018; Chiou et al., 2020). The detailed mechanism linking diabetes to TET2-mediated DNA demethylation has been unveiled by one study, which demonstrated that the hyperglycaemic condition caused a reduction in TET2 due to the inactivation of AMPK, a kinase that phosphorylates TET2 and prevents it from degradation, and epigenomic alterations occurred as a consequence of decreased TET2 (Wu et al., 2018). Autophagy-related genes, such as *BECN1*, are among the downstream targets of TET2; by regulating the expression of these genes, TET2 modulates the activity of autophagy in response to stress stimuli (Li et al., 2015; Peng et al., 2016; Yang et al., 2016). Based on the available evidence, the AMPK-TET2 axis can be viewed as a key signaling transduction pathway that rallies metabolic cues to autophagy and alters the flux of this degradation pathway.

Sodium-glucose cotransporter-2 (SGLT-2)/solute carrier family five member 2 (SLC5A2) inhibitors are a class of glucose-lowering agents that inhibit renal glucose reabsorption, thereby increasing urinary glucose excretion (Marsenic, 2009). In addition to the glucose lowering effect, it has been increasingly recognized that SGLT-2 inhibitors can exert pleiotropic effects via various mechanisms of action. One of the potential additional benefits with SGLT-2 inhibitors is improving MAFLD and preventing disease progression (Seko et al., 2017; Kuchay et al., 2018; Shibuya et al., 2018; Shimizu et al., 2019). Empagliflozin is a potent and competitive SGLT-2 inhibitor with high selectivity (Grempler et al., 2012). Empagliflozin showed promise in treating MAFLD in the E-LIFE trial, in which the combination of empagliflozin and standard therapy significantly reduced liver fat in patients with T2DM and MAFLD (Kuchay et al., 2018). In an animal model of T2DM, empagliflozin reduced hepatic steatosis, which was associated with the activation of AMPK (Kim et al., 2018). In this study, using both *in vivo* and *in vitro* models of hepatic steatosis, we investigated the effect of empagliflozin on liver fat accumulation. Furthermore, we explored underlying mechanisms and examined the roles of AMPK, TET2, and autophagy, which presumably act as the key players in the regulation of fat deposition in the liver. We demonstrated that empagliflozin could reduce hepatic steatosis, and this effect was mediated through the AMPK-TET2-autophagy pathway.

## Materials and Methods

### *In vivo* Studies

Eight-week-old male db/db mice and male db/m mice (purchased from Model Animal Research Center of Nanjing University, China) were maintained at room temperature (20–24°C) and fed with a standard chow diet *ad libitum* in environmentally controlled animal facilities at Tianjin Medical University. Db/db mice were an established animal model of T2DM and MAFLD, and it harbors a point mutation in the gene encoding a leptin receptor, which leads to fatty liver, obesity, hyperglycaemia, and other metabolic defects. After 2 weeks adaptation, db/db mice were randomly assigned into two groups and administered either empagliflozin (3.8 mg/kg/d, n = 6) or saline (same volume as used for empagliflozin, n = 6) intragastrically for eight weeks. As a control group, db/m mice (n = 6) were treated with matching volumes of saline for the same period of time. To test whether the hypoglycemic effect of empagliflozin was attributed to the genotyping in mice, another group of db/m mice (n = 6) were administered empagliflozin intragastrically for 7 days. We calculated with the appropriate dose of empagliflozin in mice based on the body surface areas of adults and mice. Body weight, blood glucose, and urine glucose were measured twice a week, over the 8 weeks course of treatment. To test whether the effects of empagliflozin were due to the decrease in blood glucose, another group of db/db mice (n = 6) were injected subcutaneously with insulin glargine. At the end of treatment, all mice were sacrificed by exsanguination under anesthesia with inhaled 5% isoflurane in room air. All animal procedures were approved by the Tianjin Medical University Experimental Animal Ethics Committee.

### Cell Culture and Treatments

HL7702 cells were cultured in RPMI-1640 (HyClone, SH30809 01) supplemented with 10% fetal bovine serum (Excell Bio) and 100U/ml penicillin (Solarbio) in a humidified incubator at 37°C with 5% CO_2_. To establish a hepatocyte steatosis model, HL7702 cells were treated with palmitic acid (PA) and high glucose (HG) when the cells reached 60%–70% confluence in six-well plates. Mannitol was used for osmolality control. After experimenting with different concentrations of PA (0, 0.1, 0.25, 0.5, 0.75 mM) and different durations (24 and 48 h), treatment with 0.5 mM PA and 25 mM glucose for 48 h was determined as the optimal condition to induce steatosis (Supplementary Figure 4B–E). Empagliflozin was applied at a concentration of 10 μM, based on results of a cell viability assay with Cell Counting Kit-8 (CCK8). To determine the role of autophagy or AMPK, HL7702 cells were induced with 0 5 mM PA and 25 mM glucose for 48 h, and then were treated with or without empagliflozin (10 μM) and with or without 3-methyladenine (3-MA, 5 mM, Selleck, TX), AICAR (10 μM, MCE), or Compound C (2 μM, Sigma). Complete culture medium was used in all experiments to avoid starvation-induced autophagy.

### Western Blot Analysis

Proteins were extracted from cells or tissues in RIPA lysis buffer supplemented with PMSF and phosphatase inhibitors and dissolved in SDS loading buffer. Equal amounts of proteins were resolved by SDS-PAGE and transferred to a nitrocellulose membrane (Millipore). After being blocked with 5% skimmed milk in 0.25% TBS-Tween (TBST), membranes were incubated with the following primary antibodies overnight at 4°C: p62 (1:2000, Abcam, ab56416), ATG5 (1:2000, Proteintech, No. 10181-2-AP), Beclin-1 (1:2000, Proteintech, No. 11306-1-AP), LC3 (1:2000, Proteintech, No. 14600-1-AP), TET2 (1:1,000, Proteintech, No. 21207-1-AP), *p*-AMPK (1:2000, Abcam, ab23875), AMPK (1:2000, Abcam, ab80039), SGLT2 (1:1,000, Proteintech, No. 24654-1-AP), SREBP-1c (1:1,000, Abcam, ab28481), PPARα (1:800, Proteintech, No. 15540-1-AP), CD36 (1:1,000, Abcam, No. 18836-1-AP), and β-actin (1:5,000, Bioworld, BS6007M). After washing with TBST, the horseradish peroxidase (HRP)-conjugated secondary antibody was incubated for 1 h at room temperature. Finally, protein bands were visualized with an ECL kit (Advansta, K-12045-D50). Quantification of each band was analyzed with ImageJ software. Proteins levels were normalized against the loading control β-actin.

### Immunofluorescence

Cells grown on coverslips were fixed in 4% paraformaldehyde for 30 min at room temperature, permeabilized with 0.1% Triton X-100 for 30 min. Cells were blocked in 5% BSA for 1 h at room temperature, washed with sterile phosphate buffer saline (PBS) for 15 min, and then incubated overnight with anti-ATG5 (1:100; Proteintech), anti-Beclin-1 (1:100; Proteintech), anti-p62 (1:200; Abcam), and anti-TET2 (1:50; Proteintech) at 4°C. Then cells were incubated with corresponding Alexa Fluor-conjugated secondary antibodies for 1 h at room temperature, washed with sterile PBS for 15 min, and counterstained with DAPI (Zhongshan Jinqiao, ZLI-9557). Fluorescence signals were detected by confocal fluorescence microscopy (Leica Microsystems).

### Hematoxylin and Eosin Staining and Immunohistochemistry

Livers were fixed in 4% paraformaldehyde, embedded in paraffin, and sectioned into four micromrter thick slices. The liver sections were deparaffinised in xylene, dehydrated in graded alcohol and washed with ddH_2_O. The liver sections were stained with hematoxylin and eosin according to the manufacturer’s protocols. For immunostaining, liver slices were blocked with 5% BSA and incubated overnight at 4°C with anti-ATG5 (1:200; Proteintech), anti-Beclin-1 (1:200; Proteintech), anti-TET2 (1:200; Proteintech), and anti-p62 (1:200; Abcam) after heat mediated antigen retrieval with citrate for 20 min. The slices were incubated with appropriate secondary antibodies at room temperature for 1 h, washed with sterile PBS for 15 min, stained with DAB, counterstained with hematoxylin, dehydrated in graded alcohol, rinsed with xylene, and mounted on slides. Images were acquired by an Olympus I3-TPC microscope.

### Oil Red O Staining

Frozen liver sections were fixed in 4% ice-cold paraformaldehyde, washed with sterile PBS for 10 min, stained with Oil Red O solution (0.5% in isopropanol, diluted with ddH_2_O in ratio of 3:2) for 20 min at room temperature, washed thrice with ddH_2_O, and finally counterstained with hematoxylin for 5 min, washed with ddH_2_O for 10 min and mounted on slides. Cells were fixed in 4% paraformaldehyde for 20 min, washed with sterile PBS for 10 min, and stained with Oil Red O for 20 min at room temperature. Finally, the cells were washed thrice with ddH_2_O. Images were taken by an Olympus I3-TPC microscope. To quantify Oil Red O staining, intracellular lipids were extracted with isopropanol and shaken for 10 min at room temperature, and the absorbance at 520 nm was measured on a monochromator microplate reader (Bio Tek).

### siRNA Transfection

Specific TET2 siRNA (5′-CCA​UCA​CAA​UUG​CUU​CUU​UTT-3′ and 5′-AAA​GAA​GCA​AUU​GUG​AUG​GTT-3′) and negative control siRNA were obtained from GenePharma. TET2 siRNA was transfected with Lipofectamine 2000 (Invitrogen, 11668019) for 6 h according to the manufacturer’s protocol. TET2 knockdown was verified by western blot (Supplementary Figure 6). After 6 h of transfection, the cells treated with PA, HG and empagliflozin for 48 h.

### Statistical Analysis

Each experiment was repeated at least three times. Statistical analysis was performed using Prism 7.0 software (GraphPad, La Jolla, CA, United States). All data were presented as the mean ± SEM from three independent experiments. Comparisons between multiple groups were analyzed by one-way ANOVA followed by the Tukey post-test. Unpaired Student’s *t*-test were used to compare the differences between two groups. Statistical significance was accepted at *p* < 0.05.

## Results

### Empagliflozin Decreases Blood Glucose and Liver Lipid Accumulation in Db/Db Mice

**FIGURE 1:**
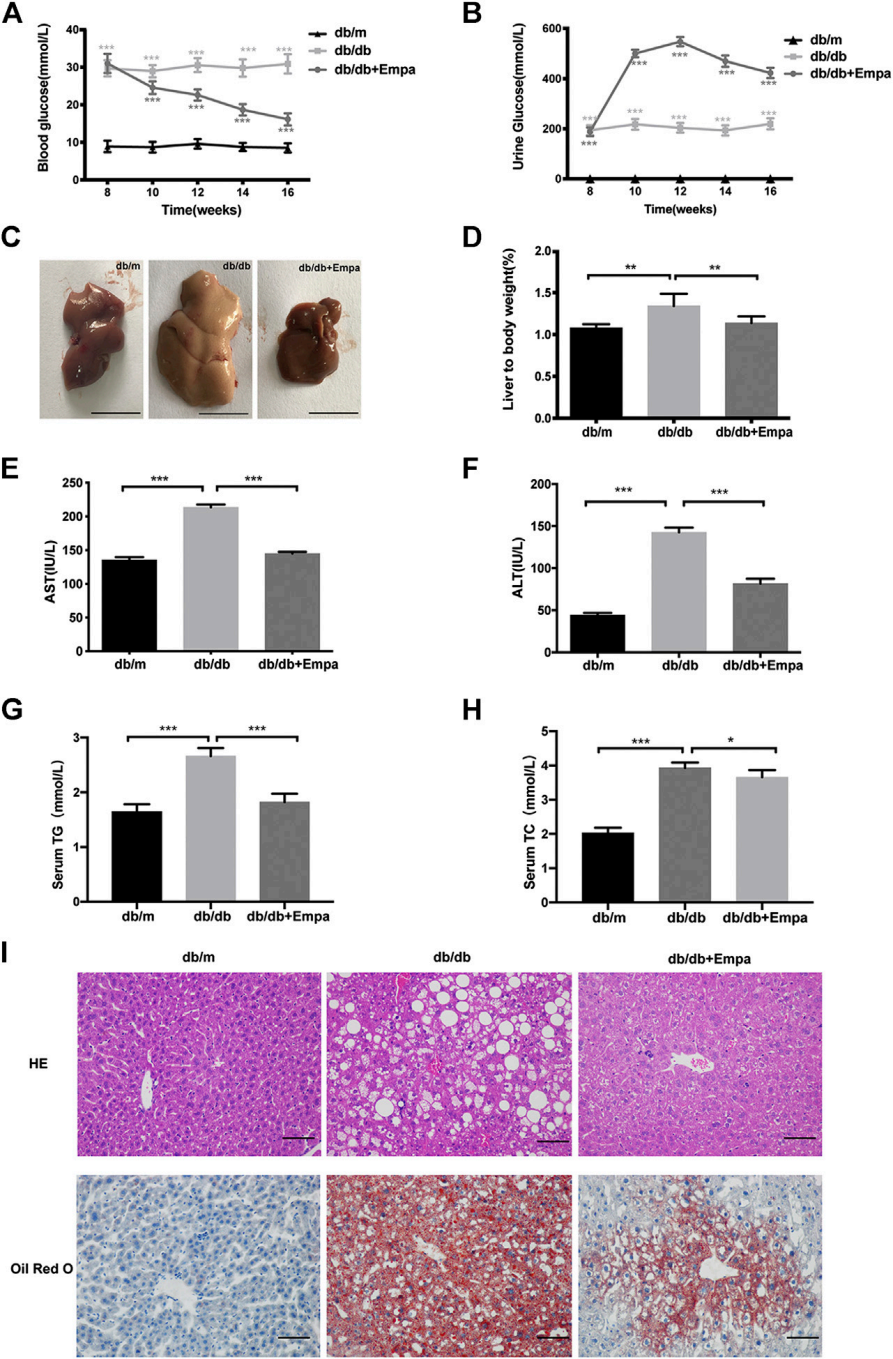
Empagliflozin ameliorates hyperglycaemia and hepatic steatosis in db/db mice. Eight-week-old male db/db mice were treated with saline or empagliflozin for eight weeks, and eight-week-old male db/m mice given no treatment were used as a control **(A)** Blood glucose and; **(B)** urine glucose concentrations over the 8-weeks treatment duration; **(C)** Representative images of mouse liver after 8 weeks treatment. Scale bar represents 1 cm **(D)** Liver to body weight and **(E–H)** serum AST, ALT, TG and TC concentrations after 8-weeks treatment **(I)** Liver histology and hepatic lipid accumulation were observed using hematoxylin-eosin staining and Oil Red O staining. The representative images are shown (Scale bar represents 200 μm). Data in **(D–F)** are presented as means ± SEM from three independent experiments. ALT, alanine aminotransferase; AST, aspartate aminotransferase; Empa, empagliflozin; HE, hematoxylin and eosin staining. *p < 0.05, **p < 0.01, ***p < 0.001.

The db/db mice exhibited hyperglycaemia and features of MAFLD including a pale and enlarged liver, increased liver to body weight ratio, elevated serum aspartate aminotransferase (AST) and alanine aminotransferase (ALT), raised serum triglyceride (TG) and total cholesterol (TC), lipid accumulation around the perisinusoidal areas in the form of macrovascular steatosis (Figure 1). Western blot results showed that CD36 and SREBP-1c were significantly increased while PPARα decreased in db/db mice compared with that of db/m mice (Supplementary Figure 1C–D), indicating that hepatic steatosis was observed in db/db mice. Empagliflozin significantly lowered blood glucose and increased urinary glucose excretion in db/db mice (Figure 1A,B). Notably, eight weeks for empagliflozin treatment, db/db mice demonstrated a normal liver phenotype and had lower serum levels of AST, ALT, TG and TC, decreased liver to body weight ratio, less hepatic lipid accumulation, improved the index for lipid metabolism (i.e., CD36, SREBP-1c, PPARα), and reduced liver weight and body weight compared with db/db mice treated with saline (Figure 1C–I and Supplementary Figure 1A–D). To test whether the hypoglycemic effect of empagliflozin was attributed to the genotyping in mice, db/m mice were treated with short-term (7 days) empagliflozin. But after short-term empagliflozin treatment, empagliflozin did not significantly alter blood glucose compared with saline in db/m mice (Supplementary Figure 1E). No adverse effects of empagliflozin were observed during treatment. These results indicated that empagliflozin had a preventative effect on the development of MAFLD in db/db mice.

### Autophagy and AMPK-TET2 Signaling Are Inhibited in Db/Db Mice and Restored by Empagliflozin

**FIGURE 2:**
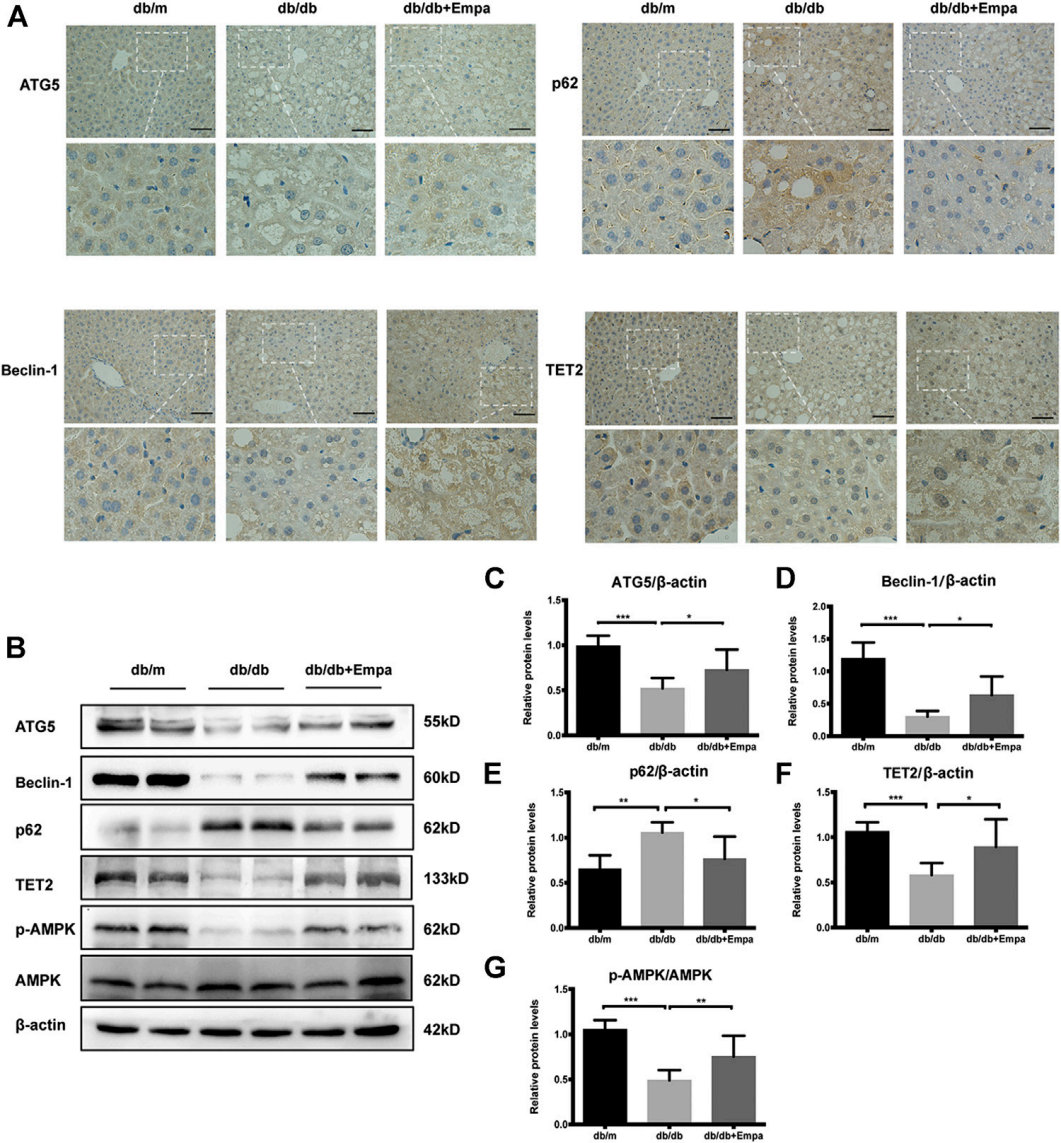
Autophagy, AMPK phosphorylation, and TET2 are decreased in db/db mice and restored by empagliflozin. Eight-week-old male db/db mice were treated with saline or empagliflozin for eight weeks, and eight-week-old male db/m mice given no treatment were used as a control **(A)** Representative immunohistochemistry images of Beclin-1, ATG5, TET2, and p62 staining in mice liver tissues. Scale bar represents 100 μm; **(B)** Western blot analyses of ATG5, Beclin-1, p62, TET2, *p*-AMPK, and AMPK with β-actin as a loading control and **(C–G)** densitometric analyses of band intensities normalized to β-actin. Data in **(C–G)** are presented as means ± SEM from three independent experiments. Empa, empagliflozin. ^*^ *p* < 0.05, **p < 0.01, ***p < 0.001.

Autophagy degrades liver lipid droplets and prevents hepatic steatosis (Ueno and Komatsu, 2017). To determine whether autophagy was suppressed in db/db mice and this activity was restored by empagliflozin, autophagy flux was evaluated with autophagy-specific markers. Although LC3II/LC3I is widely used as an indicator of autophagy, several caveats make the detection of two LC3 isoforms and the interpretation of the ratio problematic, and this ratio is not always a reliable marker of autophagy (Klionsky et al., 2016). Therefore, we used other autophagy-related proteins, p62, ATG5, and Beclin-1, to assess autophagy in subsequent experiments. Immunohistochemistry and western blot results showed that Beclin-1 and ATG5 were significantly decreased while p62 accumulated in the livers of db/db mice compared with that of db/m mice, indicating that autophagy was suppressed in db/db mice (Figure 2A–E). Empagliflozin restored autophagy activity in the livers of db/db mice, as demonstrated by the increase in Beclin-1 and ATG5 and reduction in p62 (Figure 2A–E). The livers of db/db mice also showed signs of vascular degeneration, while these histopathologic changes were ameliorated by empagliflozin (Figure 2A). To test whether the effects of empagliflozin were attributed to the decrease in blood glucose, db/db mice were treated with insulin. However, insulin treatment did not induce discernible changes in p62 and Beclin-1 compared with saline in db/db mice (Supplementary Figure 2B–D), suggesting that the effect of empagliflozin on autophagy was independent of its hypoglycaemic effect. Meanwhile we examined whether SGLT-2 was expressed in the livers of mice. Western blot results revealed that SGLT-2 was expressed in mice livers (Supplementary Figure 3).

To explore possible mechanisms by which empagliflozin affects autophagy, *p*-AMPK, AMPK, and TET2 were assessed by western blot, given that the AMPK-TET2 axis has been shown to be involved in the regulation of autophagy (Wu et al., 2018). The phosphorylation of AMPK was reduced in db/db mice, whereas it was recovered by empagliflozin treatment (Figure 2B,G). Meanwhile, the level of TET2 protein was reduced in db/db mice; however, empagliflozin treatment resulted in an increase in TET2 protein levels (Figure 2B,F).

Collectively, empagliflozin treatment enhanced autophagy, activated AMPK, and increased TET2 protein levels in db/db mice.

### Empagliflozin Reduces Lipid Accumulation and Upregulates Autophagy in a Steatosis Cell Model

**FIGURE 3:**
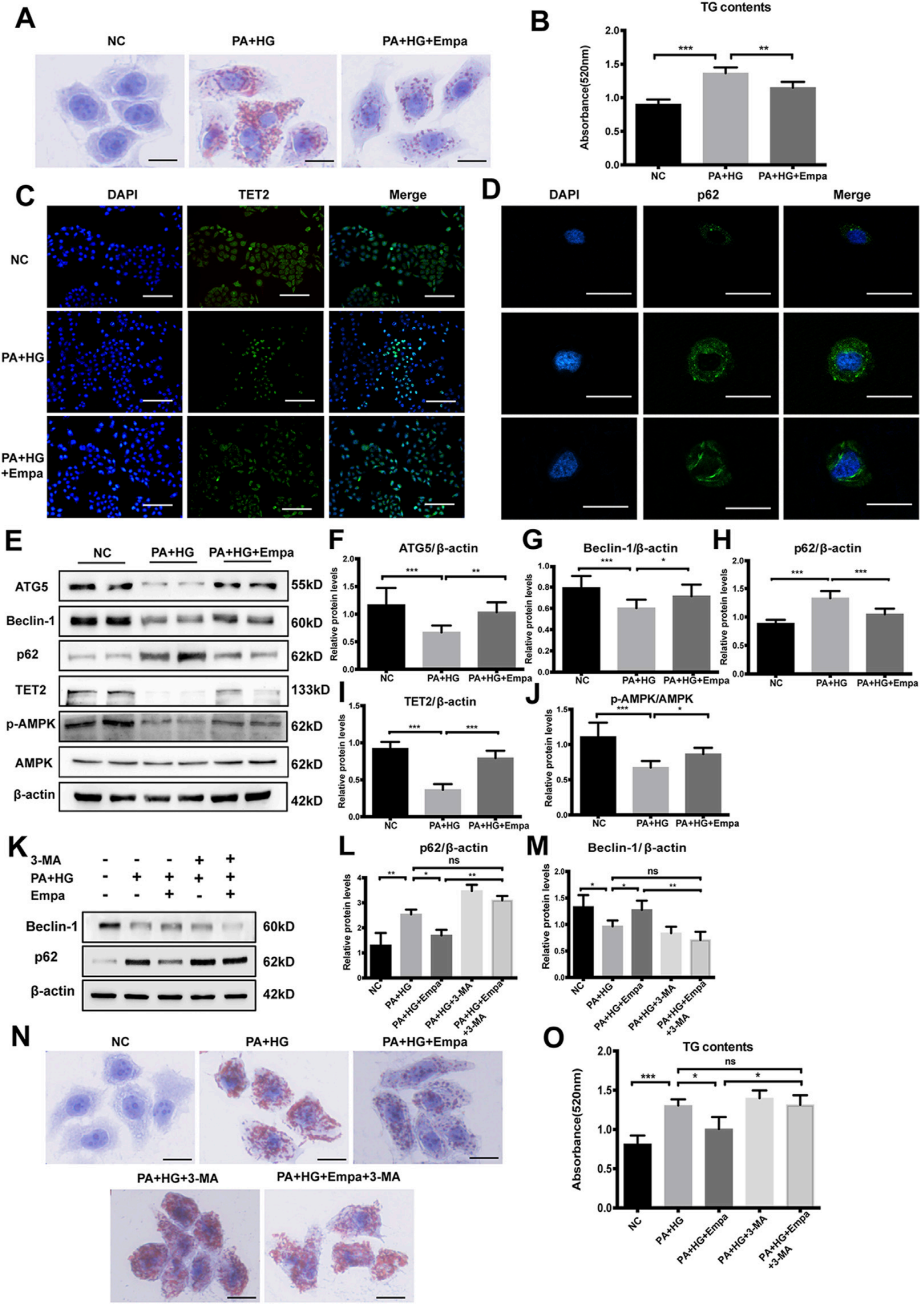
Empagliflozin ameliorates lipid accumulation and activates autophagy and the AMPK-TET2 signaling in HL7702 cells treated with PA and HG **(A–J)** HL7702 cells were treated with or without PA and HG, with or without empagliflozin **(A)** Representative images of Oil Red O staining and **(B)** quantitative analysis of TGs. Scale bar represents 30 μm. Immunofluorescence of **(C)** TET2 and **(D)** p62 in HL7702 cells. Scale bars represent 30 μm **(C)** and 100 μm **(D)**, respectively, **(E)** Western blot analyses of ATG5, Beclin-1, p62, TET2, p-AMPK, and AMPK with β-actin as a loading control and **(F–J)** densitometric analyses of band intensities normalized to β-actin **(K–O)** HL7702 cells were treated with or without PA and HG, with or without empagliflozin, in the presence or absence of 3-MA **(K)** Western blot analyses of Beclin-1 and p62 with β-actin as a loading control and **(L, M)** densitometric analyses of band intensities normalized to β-actin **(N)** Representative images of Oil Red O staining and **(O)** quantitative analyses of TGs. Data in **(B, F–J, L, M**, and **O)** are presented as means ± SEM from three independent experiments. Empa, empagliflozin; HG, high glucose; NC, negative control group; PA, palmitic acid; TG, triglyceride; 3-MA, 3-methyladenine. *p < 0.05, **p < 0.01, ***p < 0.001.

To confirm the effects of empagliflozin on autophagy and steatosis *in vitro*, we established a cell model of hepatic steatosis and treated cells with empagliflozin. Firstly, we tested the expression of SGLT-2 in HL7702 cells (Supplementary Figure 3). Then in order to determine the optimal condition to induce steatosis, HL7702 cells were treated with ascending concentrations of PA (0, 0.01, 0.25, 0.5, 0.75 mM) for different durations (24 and 48 h). Based on the accumulation of p62, the reduction in TET2, and lipid deposition, 0.5 mM PA and 25 mM glucose for 48 h was selected as the optimal treatment regimen (Supplementary Figure 4B–E). Furthermore, there was no significant difference between mannitol and normal control (NC), excluding the interference of hyperosmosis (Supplementary Figure 5A,B). Lipid deposits were markedly elevated in this hepatocyte steatosis model, demonstrated by the increase in Oil Red O staining (Figure 3A) and TG level (Figure 3B). Treatment with empagliflozin significantly attenuated lipid accumulation in these cells (Figure 3A,B).

Then we evaluated autophagy flux in the hepatocyte steatosis model. Immunofluorescence and western blot results showed that under PA and HG-induced steatosis, cellular levels of ATG5 and Beclin-1 were decreased, and p62 was increased (Figure 3D–H). Empagliflozin treatment reversed the effects induced by PA and HG, increasing ATG5 and Beclin-1 and reducing p62 (Figure 3D–H). Consistent with NC group, cells exposed to PA and HG showed reduced *p*-AMPK and TET2, but empagliflozin significantly up-regulated AMPK-activity and TET2 expression (Figure 3C,E,I,J). However, empagliflozin treatment had no effect in NC group (Supplementary Figure 5A, 5B). These data demonstrated that empagliflozin increased AMPK phosphorylation and TET2, induced autophagy, and reduced lipid accumulation in the hepatocyte steatosis model.

To test whether autophagy mediates the preventative effect of empagliflozin on lipid accumulation, we blocked autophagy with its inhibitor, 3-MA. While PA and HG significantly increased intracellular lipid accumulation, empagliflozin decreased lipid contents in the cells (Figure 3N,O). However, in the presence of 3-MA, empagliflozin was no longer able to reduce lipid accumulation in hepatocytes exposed to PA and HG (Figure 3N,O). These results indicated that autophagy activity was required for the reduction of lipid accumulation by empagliflozin.

### Empagliflozin Elevates TET2 and Autophagy Through AMPK

**FIGURE 4:**
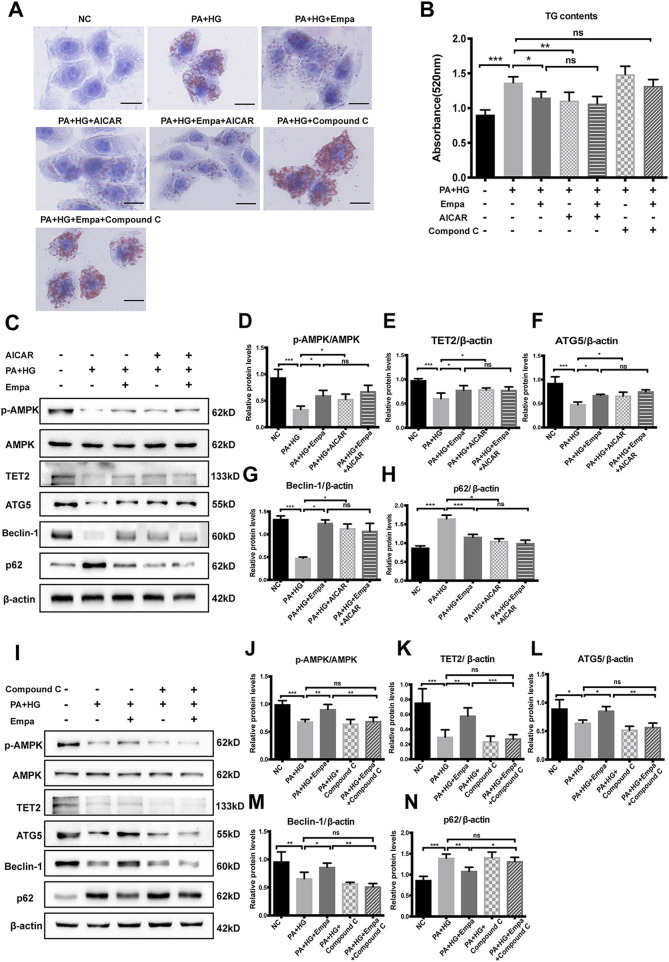
The increase in TET2 and autophagy and reduction in lipid accumulation by empagliflozin are dependent on AMPK activation. HL7702 cells were treated with or without PA and HG, with or without empagliflozin, in the presence or absence of AICAR or compound C **(A)** Representative images of oil red O staining and **(B)** quantitative analysis of TGs. Scale bar represents 30 μm **(C, I)** Western blot analyses of p-AMPK, AMPK, TET2, ATG5, Beclin-1, and p62 with β-actin as a loading control and **(D–H, J–N)** densitometric analyses of band intensities normalized to β-actin. Data in **(B, D–H, J–N)** are presented as means ± SEM from three independent experiments. Empa, empagliflozin; HG, high glucose; NC, negative control group; ns, not significant; PA, palmitic acid; TG, triglyceride. *p < 0.05, **p < 0.01, ***p < 0.001.

Active AMPK phosphorylates TET2 and protects it from degradation in peripheral blood mononuclear cells (Wu et al., 2018). We hypothesized that empagliflozin treatment led to the activation of AMPK, thereby stabilizing TET2 and increasing its intracellular level. To test this hypothesis, we treated steatotic hepatocytes with the AMPK activator AICAR or its inhibitor, Compound C. The reagent used for testing was consistent with other studies (Koyani et al., 2020; Wang et al., 2020). Similar to empagliflozin, AICAR significantly reduced hepatocellular lipid content; in contrast, Compound C exacerbated lipid accumulation in the hepatocyte steatosis model (Figure 4A,B). More importantly, empagliflozin did not alleviate PA and HG-induced steatosis in the presence of Compound C (Figure 4A,B). Both empagliflozin and AICAR increased TET2 in the hepatocyte steatosis model (Figure 4C,E). With regard to autophagy, both empagliflozin and AICAR significantly decreased p62 while increased Beclin-1 and ATG5 (Figure 4C,F–H). In contrast, Compound C decreased the levels of TET2, ATG5, and Beclin-1 and increased p62 in the hepatocyte steatosis model (Figure 4I,K–N). Notably, in the presence of AICAR or Compound C, empagliflozin treatment had no additional effects on TET2 and the autophagy markers (Figure 4C,E–I,K–N). These results suggested that AMPK was involved in the regulation of TET2, autophagy, and lipid deposition under empagliflozin treatment.

### TET2 Is Required for Autophagy Induction and Reduction of Lipid Accumulation by Empagliflozin

**FIGURE 5:**
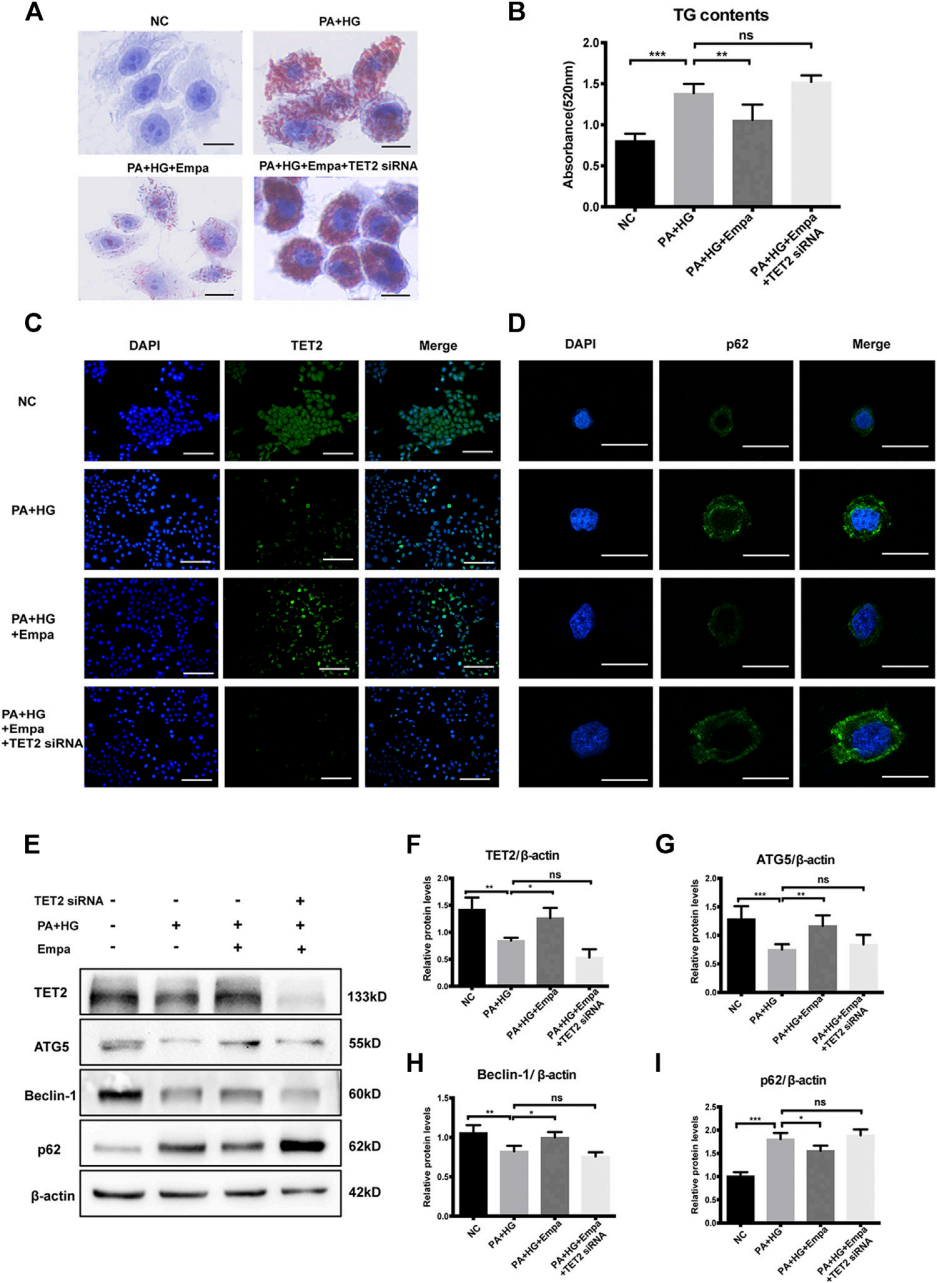
TET2 is required for empagliflozin-induced autophagy. HL7702 cells were transfected with or without *TET2* siRNA and treated with or without PA and HG, with or without empagliflozin **(A)** Representative images of Oil Red O staining and **(B)** quantitative analysis of TGs. Scale bar represents 30 μm. Immunofluorescence of **(C)** TET2 and **(D)** p62. Scale bars represent 30 μm **(C)** and 100 μm **(D)**, respectively **(E)** Western blot analyses of TET2, ATG5, Beclin-1, and p62 with β-actin as a loading control and **(F–I)** densitometric analyses of band intensities normalized to β-actin. Data in **(B)** and **(F–I)** are presented as means ± SEM from three independent experiments. Empa, empagliflozin; HG, high glucose; NC, negative control group; PA, palmitic acid; TG, triglyceride. *p < 0.05, **p < 0.01, ***p < 0.001.

To investigate the potential role of TET2 in inducing autophagy and reducing lipid content, siRNA directed against TET2 was transfected into HL7702 cells. The preventive effect of empagliflozin on PA and HG-induced steatosis was abolished when TET2 was knocked-down (Figure 5A,B). Also, under the depletion of TET2, autophagy was no longer activated by empagliflozin in the hepatocyte steatosis model, based on the levels of Beclin-1, ATG5, and p62 (Figure 5C–I). We concluded that TET2 contributed to empagliflozin-induced autophagy and the regulation of lipid levels in hepatocytes.

## Discussion

Patients with T2DM are at high risk of developing MAFLD due to systemic metabolic dyshomeostasis (Tilg et al., 2017). Preventing and treating MAFLD in patients with T2DM is critical since it may progress into advanced liver diseases that are associated with high morbidity and mortality. In the current study, we demonstrated that empagliflozin could reduce hepatic lipid accumulation and prevent hepatic steatosis both *in vitro* and *in vivo*. The data indicated that empagliflozin might exert this effect via activation of autophagy through the AMPK-TET2 signaling.

SGLT-2 inhibitors are commonly used to treat T2DM, and they lower blood glucose by inhibiting glucose reabsorption in the kidney and promoting urinary glucose excretion. In addition to the sustained hypoglycaemic effect, SGLT-2 inhibitors are also able to reduce body weight and abdominal fat (Cefalu et al., 2013). It was demonstrated *in vivo* that tofogliflozin induced a switch from carbohydrate oxidation to fatty acid oxidation, which might contribute to the decrease in fat accumulation in the adipose tissue and liver (Suzuki et al., 2014). In patients with T2DM and MAFLD, luseogliflozin reduced liver fat and body weight (Shibuya et al., 2018), and similarly, dapagliflozin ameliorated hepatic steatosis and fibrosis (Shimizu et al., 2019). The E-LIFT trial showed that empagliflozin combined with standard therapy reduced liver fat in patients with T2DM and MAFLD (Kuchay et al., 2018). In db/db mice, empagliflozin prevented hepatic steatosis, but insulin alone elevated TG in the liver (Katsiki et al., 2019). In our study, empagliflozin treatment reduced blood glucose, body weight, hepatic TG content, and hepatic steatosis and enhanced urinary glucose excretion in db/db mice. In the PA and HG-induced steatosis cell model, intracellular content of lipid and TGs was significantly decreased after treatment with empagliflozin. These results are consistent with previous reports (Jojima et al., 2016; Bajaj et al., 2018; Lai et al., 2020). In comparison, insulin injection did not improve MAFLD despite the glucose-lowering effect. Our results and the available preclinical and clinical evidence point to the potential benefit of preventing fatty liver and improving MAFLD with empagliflozin in patients with T2DM.

Autophagy contributes to lipid metabolic balance and alleviates liver damage and inflammation (Liu and Czaja, 2013; Sinha et al., 2014). Suppressed autophagy may underlie the pathogenesis and progression of MAFLD (Madrigal-Matute and Cuervo, 2016). Particularly, lipophagy is a subtype of autophagy where intracellular lipid droplets are engulfed by the autophagosome, transported to and subsequently degraded in the lysosome (Sathyanarayan et al., 2017). Inhibition of this degradation process is associated with ectopic lipid deposition in the liver and hepatic steatosis (Liu and Czaja, 2013). Steatosis of hepatocytes is a hallmark of MAFLD, and with the build-up of fat in liver, inflammation, cirrhosis, and liver damage may ensue. Diabetic mice developed steatohepatitis, in parallel with inhibition of autophagy in the liver (Zhang et al., 2015). Based on the role of autophagy in maintaining lipid balance in the liver, it can be postulated that activating autophagy is a potential approach to treating MAFLD. In our study, empagliflozin treatment led to activation of autophagy in both db/db mice and a hepatocyte steatosis model, based on alterations of autophagy markers including p62, Beclin-1, and ATG5. Nonetheless, the ratio of LC3II/LC3I changed in an opposing manner to Beclin-1 and ATG5 in db/db mice. The accumulation of LC3-II relative to LC3I under autophagy inhibition was also observed in other studies (Gonzalez-Rodriguez et al., 2014; Wang et al., 2015a)and a guideline on the assays for monitoring autophagy warned against relying on LC3II/LC3I as an indicator of autophagy flux (Klionsky et al., 2016).

We explored the potential mechanism mediating autophagy activation under empagliflozin treatment. AMPK is a serine/threonine protein kinase that regulates a variety of cellular processes, including metabolic homeostasis, cell proliferation, and cell death. The role of AMPK in modulating autophagy and its clinical implications have been elucidated (Zaouali et al., 2013; Oh et al., 2016). In diabetic mice, empagliflozin treatment activated AMPK, which led to inhibition of mitochondrial fissions and alleviation of cardiac microvascular injury (Zhou et al., 2018). A novel SGLT-2 inhibitor, NGI001, increased AMPK phosphorylation and ameliorated lipid accumulation and inflammation in human hepatocytes (Chiang et al., 2020). Consistently, in the present study, under steatosis-inducing conditions, empagliflozin elevated AMPK phosphorylation, resulting in an increase in autophagy and reduction in lipid deposition in hepatocytes both *in vivo* and *in vitro*.

TET2 is an α-ketoglutarate- and Fe^2+^-dependent dioxygenase that mediates DNA demethylation by catalyzing the oxidation of 5-mC to 5-hmC.8 As a tumor suppressor gene, TET2 was first discovered in acute myeloid leukemia (Abdel-Wahab and Levine, 2013), and its mutation was closely related to hematopoietic malignancy (Cimmino et al., 2015). The epigenomic regulation of gene expression by TET2 and its involvement in carcinogenesis have been studied in various cancers, but its relationship with metabolic disorders has only emerged recently. Disturbance in the DNA methylation-demethylation balance plays an important role in the pathogenesis of metabolic disorders (Feinberg et al., 2010). A recent study demonstrated that TET2 functioned as an epigenetic regulator of PPARG target genes, and it was required for Rosiglitazone-mediated insulin sensitization in adipocytes (Bian et al., 2018). Under hyperglycaemia, more TET2 proteins were degraded due to the loss of its phosphorylation by AMPK, resulting in epigenomic reprogramming (Wu et al., 2018). TET2 may function as a master of autophagy by regulating the expression of Beclin-1, and the activity of autophagy was suppressed if the level of TET2 was reduced in the cell (Peng et al., 2016; Yang et al., 2016). We showed that the TET2 might be an intermediate regulator of the AMPK-autophagy pathway in hepatocytes. Empagliflozin, by activating AMPK, preserves the intracellular level of TET2 protein, which in turn induces autophagy; through the AMPK-TET2-autophagy signaling, empagliflozin reduces hepatic lipid accumulation and has potential to improve MAFLD. One study reported that empagliflozin caused modest AMPK activation at doses well above its peak plasma concentrations (Hawley et al., 2016). We posit that this inconsistency may be due to differences in cell models (HEK-293 vs HL7702) and treatment regimens (treating with empagliflozin only vs treating with empagliflozin after induction with PA and HG). However, the upstream mechanism by which empagliflozin activates AMPK remains elusive, given that the target of empagliflozin, SLC5A2, is rarely expressed in hepatocytes. Further studies are needed to reveal how empagliflozin regulates AMPK, whether additional mechanisms exist for its regulation of autophagy and lipid content in hepatocytes, and the clinical relevance of these effects.

In summary, our results demonstrate that empagliflozin, through the AMPK-TET2-autophagy pathway, reduces lipid accumulation and alleviates hepatic steatosis in *vitro* and *in vivo* models. Based on these findings and previous clinical evidence, empagliflozin could have potential to improve outcomes for patients with T2DM and MAFLD.

## Data Availability Statement

The original contributions presented in the study are included in the article/Supplementary Material, further inquiries can be directed to the corresponding authors.

## Ethics Statement

The animal study was reviewed and approved by Tianjin Medical University Experimental Animal Ethics Committee, China.

## Author Contributions

TL: Methodology, Validation, Formal analysis, Visualization, Writing–Original Draft. TF: Methodology, Validation, Formal analysis, Writing–original draft. LX: Formal analysis, Writing–review and editing, Investigation. XL: Formal analysis, Writing–review and editing, Investigation. XL: Investigation, Resources. MX: Investigation, Resources. XY: Resources. BS: Conceptualization, Writing–Review and Editing, Supervision, Project administration. LC: Conceptualization, Writing–Review and Editing, Supervision, Project administration. BS is the guarantor of this work and, as such, had full access to all the data in the study and takes responsibility for the integrity of the data and the accuracy of the data analysis.

## Funding

This work was financially supported by grants from the Natural Science Foundation of Tianjin (18JCYBJC26100, 18JCZDJC35500 and 18JCYBJC25000) and the National Natural Science Foundation of China (81970697). The funders played no role in study design, data collection or interpretation, the writing of the manuscript, or the decision to submit the manuscript for publication.

## Conflict of Interest

The authors declare that the research was conducted in the absence of any commercial or financial relationships that could be construed as a potential conflict of interest.

## References

[B1] Abdel-Wahab O. Levine R. L. (2013). Mutations in epigenetic modifiers in the pathogenesis and therapy of acute myeloid leukemia. Blood. 121, 3563–3572. 10.1182/blood-2013-01-451781 23640996 PMC3643757

[B2] Bajaj H. S. Brown R. E. Bhullar L. Sohi N. Kalra S. Aronson R. (2018). SGLT2 inhibitors and incretin agents: associations with alanine aminotransferase activity in type 2 diabetes. Diabetes Metab. 44, 493–499. 10.1016/j.diabet.2018.08.001 30149145

[B3] Bian F. Ma X. Villivalam S. D. You D. Choy L. R. Paladugu A. (2018). TET2 facilitates PPARgamma agonist-mediated gene regulation and insulin sensitization in adipocytes. Metabolism. 89, 39–47. 10.1016/j.metabol.2018.08.006 30193945 PMC6221917

[B4] Cefalu W. T. Leiter L. A. Yoon K. H. Arias P. Niskanen L. Xie J. (2013). Efficacy and safety of canagliflozin versus glimepiride in patients with type 2 diabetes inadequately controlled with metformin (CANTATA-SU): 52 week results from a randomised, double-blind, phase 3 non-inferiority trial. Lancet. 382, 941–950. 10.1016/s0140-6736(13)60683-2 23850055

[B5] Chiang H. Lee J. C. Huang H. C. Huang H. Liu H. K. Huang C. (2020). Delayed intervention with a novel SGLT2 inhibitor NGI001 suppresses diet-induced metabolic dysfunction and non-alcoholic fatty liver disease in mice. Br. J. Pharmacol. 177, 239–253. 10.1111/bph.14859 31497874 PMC6989948

[B6] Chiou J. T. Huang C. H. Lee Y. C. Wang L. J. Shi Y. J. Chen Y. J. (2020). Compound C induces autophagy and apoptosis in parental and hydroquinone-selected malignant leukemia cells through the ROS/p38 MAPK/AMPK/TET2/FOXP3 axis. Cell Biol. Toxicol. 36 (4), 315–331. 10.1007/s10565-019-09495-3 31900833

[B7] Cimmino L. Dawlaty M. M. Ndiaye-Lobry D. Yap Y. S. Bakogianni S. Yu Y. (2015). TET1 is a tumor suppressor of hematopoietic malignancy. Nat. Immunol. 16, 653–662. 10.1038/ni.3148 25867473 PMC4545281

[B8] Eslam M. Newsome P. N. Sarin S. K. Anstee Q. M. Targher G. Romero-Gomez M. (2020). A new definition for metabolic dysfunction-associated fatty liver disease: an international expert consensus statement. J. Hepatol. 73, 202–209. 10.1016/j.jhep.2020.03.039 32278004

[B9] Feinberg A. P. Irizarry R. A. Fradin D. Aryee M. J. Murakami P. Aspelund T. (2010). Personalized epigenomic signatures that are stable over time and covary with body mass index. Sci. Transl. Med. 2, 49ra67. 10.1126/scitranslmed.3001262 PMC3137242 20844285

[B10] Fukuo Y. Yamashina S. Sonoue H. Arakawa A. Nakadera E. Aoyama T. (2014). Abnormality of autophagic function and cathepsin expression in the liver from patients with non-alcoholic fatty liver disease. Hepatol. Res. 44, 1026–1036. 10.1111/hepr.12282 24299564

[B11] Gonzalez-Rodriguez A. Mayoral R. Agra N. Valdecantos M. P. Pardo V. Miquilena-Colina M. E. (2014). Impaired autophagic flux is associated with increased endoplasmic reticulum stress during the development of NAFLD. Cell Death Dis. 5, e1179. 10.1038/cddis.2014.162 24743734 PMC4001315

[B12] Grempler R. Thomas L. Eckhardt M. Himmelsbach F. Sauer A. Sharp D. E. (2012). Empagliflozin, a novel selective sodium glucose cotransporter-2 (SGLT-2) inhibitor: characterisation and comparison with other SGLT-2 inhibitors. Diabetes Obes. Metabol. 14, 83–90. 10.1111/j.1463-1326.2011.01517.x 21985634

[B13] Hawley S. A. Ford R. J. Smith B. K. Gowans G. J. Mancini S. J. Pitt R. D. (2016). The Na+/Glucose cotransporter inhibitor canagliflozin activates AMPK by inhibiting mitochondrial function and increasing cellular AMP levels. Diabetes. 65, 2784–2794. 10.2337/db16-0058 27381369 PMC5689380

[B14] Jojima T. Tomotsune T. Iijima T. Akimoto K. Suzuki K. Aso Y. (2016). Empagliflozin (an SGLT2 inhibitor), alone or in combination with linagliptin (a DPP-4 inhibitor), prevents steatohepatitis in a novel mouse model of non-alcoholic steatohepatitis and diabetes. Diabetol. Metab. Syndrome. 8, 45. 10.1186/s13098-016-0169-x PMC4960737 27462372

[B15] Kanwal F. Kramer J. R. Li L. Dai J. Natarajan Y. Yu X. (2019). Effect of metabolic traits on the risk of cirrhosis and hepatocellular cancer in nonalcoholic fatty liver disease. Hepatology. 10.1002/hep.31014 31675427

[B16] Katsiki N. Perakakis N. Mantzoros C. (2019). Effects of sodium-glucose co-transporter-2 (SGLT2) inhibitors on non-alcoholic fatty liver disease/non-alcoholic steatohepatitis: ex quo et quo vadimus?. Metabolism. 98, iii–ix. 10.1016/j.metabol.2019.07.009 31301336

[B17] Kim J. W. Lee Y. J. You Y. H. Moon M. K. Yoon K. H. Ahn Y. B. (2018). Effect of sodium-glucose cotransporter 2 inhibitor, empagliflozin, and alpha-glucosidase inhibitor, voglibose, on hepatic steatosis in an animal model of type 2 diabetes. J. Cell. Biochem. 120, 8534–8546. 10.1002/jcb.28141 30474134

[B18] Klionsky D. J. Abdelmohsen K. Abe A. Abedin M. J. Abeliovich H. Acevedo Arozena A. (2016). Guidelines for the use and interpretation of assays for monitoring autophagy. Autophagy. 12, 1–222. 10.1080/15548627.2015.1100356 26799652 PMC4835977

[B19] Koyani C. N. Plastira I. Sourij H. Hallström S. Schmidt A. Rainer P. P. (2020). Empagliflozin protects heart from inflammation and energy depletion via AMPK activation. Pharmacol. Res. 158, 104870. 10.1016/j.phrs.2020.104870 32434052

[B20] Kuchay M. S. Krishan S. Mishra S. K. Farooqui K. J. Singh M. K. Wasir J. S. (2018). Effect of empagliflozin on liver fat in patients with type 2 diabetes and nonalcoholic fatty liver disease: a randomized controlled trial (E-LIFT trial). Diabetes Care. 41, 1801–1808. 10.2337/dc18-0165 29895557

[B21] Lai L. L. Vethakkan S. R. Nik Mustapha N. R. Mahadeva S. Chan W. K. (2020). Empagliflozin for the treatment of nonalcoholic steatohepatitis in patients with type 2 diabetes mellitus. Dig. Dis. Sci. 65, 623–631. 10.1007/s10620-019-5477-1 30684076

[B22] Lee A. Sun Y. Lin T. Song N. J. Mason M. L. Leung J. H. (2020). Amino acid-based compound activates atypical PKC and leptin receptor pathways to improve glycemia and anxiety like behavior in diabetic mice. Biomaterials. 239, 119839. 10.1016/j.biomaterials.2020.119839 32065973 PMC7085115

[B23] Li G. Peng J. Liu Y. Li X. Yang Q. Li Y. (2015). Oxidized low-density lipoprotein inhibits THP-1-derived macrophage autophagy via TET2 down-regulation. Lipids. 50, 177–183. 10.1007/s11745-014-3977-5 25503193

[B24] Liu K. Czaja M. J. (2013). Regulation of lipid stores and metabolism by lipophagy. Cell Death Differ. 20, 3–11. 10.1038/cdd.2012.63 22595754 PMC3524634

[B25] Madrigal-Matute J. Cuervo A. M. (2016). Regulation of liver metabolism by autophagy. Gastroenterology. 150, 328–339. 10.1053/j.gastro.2015.09.042 26453774 PMC4728051

[B26] Marsenic O. (2009). Glucose control by the kidney: an emerging target in diabetes. Am. J. Kidney Dis. 53, 875–883. 10.1053/j.ajkd.2008.12.031 19324482

[B27] Multhaup M. L. Seldin M. M. Jaffe A. E. Lei X. Kirchner H. Mondal P. (2015). Mouse-human experimental epigenetic analysis unmasks dietary targets and genetic liability for diabetic phenotypes. Cell Metabol. 21, 138–149. 10.1016/j.cmet.2014.12.014 PMC4340475 25565211

[B28] Oh T. S. Cho H. Cho J. H. Yu S. W. Kim E. K. (2016). Hypothalamic AMPK-induced autophagy increases food intake by regulating NPY and POMC expression. Autophagy. 12, 2009–2025. 10.1080/15548627.2016.1215382 27533078 PMC5103348

[B29] Pan W. Zhu S. Qu K. Meeth K. Cheng J. He K. (2017). The DNA methylcytosine dioxygenase Tet2 sustains immunosuppressive function of tumor-infiltrating myeloid cells to promote melanoma progression. Immunity. 47, 284–297. 10.1016/j.immuni.2017.07.020 28813659 PMC5710009

[B30] Peng J. Yang Q. Li A. F. Li R. Q. Wang Z. Liu L. S. (2016). Tet methylcytosine dioxygenase 2 inhibits atherosclerosis via upregulation of autophagy in ApoE-/- mice. Oncotarget. 7, 76423–76436. 10.18632/oncotarget.13121 27821816 PMC5363520

[B31] Sathyanarayan A. Mashek M. T. Mashek D. G. (2017). ATGL promotes autophagy/lipophagy via SIRT1 to control hepatic lipid droplet catabolism. Cell Rep. 19, 1–9. 10.1016/j.celrep.2017.03.026 28380348 PMC5396179

[B32] Seko Y. Sumida Y. Tanaka S. Mori K. Taketani H. Ishiba H. (2017). Effect of sodium glucose cotransporter 2 inhibitor on liver function tests in Japanese patients with non-alcoholic fatty liver disease and type 2 diabetes mellitus. Hepatol. Res. 47, 1072–1078. 10.1111/hepr.12834 27925353

[B33] Shibuya T. Fushimi N. Kawai M. Yoshida Y. Hachiya H. Ito S. (2018). Luseogliflozin improves liver fat deposition compared to metformin in type 2 diabetes patients with non-alcoholic fatty liver disease: a prospective randomized controlled pilot study. Diabetes Obes. Metabol. 20, 438–442. 10.1111/dom.13061 28719078

[B34] Shimizu M. Suzuki K. Kato K. Jojima T. Iijima T. Murohisa T. (2019). Evaluation of the effects of dapagliflozin, a sodium-glucose co-transporter-2 inhibitor, on hepatic steatosis and fibrosis using transient elastography in patients with type 2 diabetes and non-alcoholic fatty liver disease. Diabetes Obes. Metabol. 21, 285–292. 10.1111/dom.13520 30178600

[B35] Singh R. Kaushik S. Wang Y. Xiang Y. Novak I. Komatsu M. (2009). Autophagy regulates lipid metabolism. Nature. 458, 1131–1135. 10.1038/nature07976 19339967 PMC2676208

[B36] Sinha R. A. Farah B. L. Singh B. K. Siddique M. M. Li Y. Wu Y. (2014). Caffeine stimulates hepatic lipid metabolism by the autophagy-lysosomal pathway in mice. Hepatology. 59, 1366–1380. 10.1002/hep.26667 23929677

[B37] Suzuki M. Takeda M. Kito A. Fukazawa M. Yata T. Yamamoto M. (2014). Tofogliflozin, a sodium/glucose cotransporter 2 inhibitor, attenuates body weight gain and fat accumulation in diabetic and obese animal models. Nutr. Diabetes. 4, e125. 10.1038/nutd.2014.20 25000147 PMC5189930

[B38] Tahiliani M. Koh K. P. Shen Y. Pastor W. A. Bandukwala H. Brudno Y. (2009). Conversion of 5-methylcytosine to 5-hydroxymethylcytosine in mammalian DNA by MLL partner TET1. Science. 324, 930–935. 10.1126/science.1170116 19372391 PMC2715015

[B39] Targher G. Lonardo A. Byrne C. D. (2018). Nonalcoholic fatty liver disease and chronic vascular complications of diabetes mellitus. Nat. Rev. Endocrinol. 14, 99–114. 10.1038/nrendo.2017.173 29286050

[B40] Tilg H. Moschen A. R. Roden M. (2017). NAFLD and diabetes mellitus. Nat. Rev. Gastroenterol. Hepatol. 14, 32–42. 10.1038/nrgastro.2016.147 27729660

[B41] Ueno T. Komatsu M. (2017). Autophagy in the liver: functions in health and disease. Nat. Rev. Gastroenterol. Hepatol. 14, 170–184. 10.1038/nrgastro.2016.185 28053338

[B42] Volkmar M. Dedeurwaerder S. Cunha D. A. Ndlovu M. N. Defrance M. Deplus R. (2012). DNA methylation profiling identifies epigenetic dysregulation in pancreatic islets from type 2 diabetic patients. EMBO J. 31, 1405–1426. 10.1038/emboj.2011.503 22293752 PMC3321176

[B43] von Meyenn F. Iurlaro M. Habibi E. Liu N. Q. Salehzadeh-Yazdi A. Santos F. (2016). Impairment of DNA methylation maintenance is the main cause of global demethylation in naive embryonic stem cells. Mol. Cell. 62, 848–861. 10.1016/j.molcel.2016.04.025 27237052 PMC4914828

[B44] Wang C. He H. Liu G. Ma H. Li L. Jiang M. (2020). DT-13 induced apoptosis and promoted differentiation of acute myeloid leukemia cells by activating AMPK-KLF2 pathway. Pharmacol. Res. 158, 104864. 10.1016/j.phrs.2020.104864 32416217

[B45] Wang L. Liu X. Nie J. Zhang J. Kimball S. R. Zhang H. (2015a). ALCAT1 controls mitochondrial etiology of fatty liver diseases, linking defective mitophagy to steatosis. Hepatology. 61, 486–496. 10.1002/hep.27420 25203315 PMC4303512

[B46] Wang Y. Xiao M. Chen X. Chen L. Xu Y. Lv L. (2015b). WT1 recruits TET2 to regulate its target gene expression and suppress leukemia cell proliferation. Mol. Cell. 57, 662–673. 10.1016/j.molcel.2014.12.023 25601757 PMC4336627

[B47] Wu D. Hu D. Chen H. Shi G. Fetahu I. S. Wu F. (2018). Glucose-regulated phosphorylation of TET2 by AMPK reveals a pathway linking diabetes to cancer. Nature. 559, 637–641. 10.1038/s41586-018-0350-5 30022161 PMC6430198

[B48] Yang L. Li P. Fu S. Calay E. S. Hotamisligil G. S. (2010). Defective hepatic autophagy in obesity promotes ER stress and causes insulin resistance. Cell Metabol. 11, 467–478. 10.1016/j.cmet.2010.04.005 PMC2881480 20519119

[B49] Yang Q. Li X. Li R. Peng J. Wang Z. Jiang Z. (2016). Low shear stress inhibited endothelial cell autophagy through TET2 downregulation. Ann. Biomed. Eng. 44, 2218–2227. 10.1007/s10439-015-1491-4 26493943

[B50] Zaouali M. A. Boncompagni E. Reiter R. J. Bejaoui M. Freitas I. Pantazi E. (2013). AMPK involvement in endoplasmic reticulum stress and autophagy modulation after fatty liver graft preservation: a role for melatonin and trimetazidine cocktail. J. Pineal Res. 55, 65–78. 10.1111/jpi.12051 23551302

[B51] Zhang Q. Li Y. Liang T. Lu X. Zhang C. Liu X. (2015). ER stress and autophagy dysfunction contribute to fatty liver in diabetic mice. Int. J. Biol. Sci. 11, 559–568. 10.7150/ijbs.10690 25892963 PMC4400387

[B52] Zhou H. Wang S. Zhu P. Hu S. Chen Y. Ren J. (2018). Empagliflozin rescues diabetic myocardial microvascular injury via AMPK-mediated inhibition of mitochondrial fission. Redox Biol. 15, 335–346. 10.1016/j.redox.2017.12.019 29306791 PMC5756062

