# Corrigendum: Empagliflozin alleviates hepatic steatosis by activating the AMPK-TET2-autophagy pathway *in vivo* and *in vitro*

**DOI:** 10.3389/fphar.2025.1478437

**Published:** 2025-01-28

**Authors:** Ting Li, Ting Fang, Linxin Xu, Xiangyang Liu, Xiaoyu Li, Mei Xue, Xiaochen Yu, Bei Sun, Liming Chen

**Affiliations:** NHC Key Laboratory of Hormones and Development, Tianjin Key Laboratory of Metabolic Diseases, Chu Hsien-I Memorial Hospital and Tianjin Institute of Endocrinology, Tianjin Medical University, Tianjin, China

**Keywords:** empagliflozin, autophagy, diabetes, metabolic associated fatty liver disease, lipid accumulation, ten-eleven translocation 2

In the published article, there was an error in Figure 3 as published. The merge of the PA+HG+Empa group (Figure 3D) was presented correctly, but the DAPI of PA + HG + Empa group (Figure 3D) was presented incorrectly. When the authors were preparing the DAPI of PA + HG + Empa group in Figure 3D, they mistakenly copied the image of the DAPI of NC group in **Figure 5D**. The corrected Figure 3 and its caption appear below.

**FIGURE 3:**
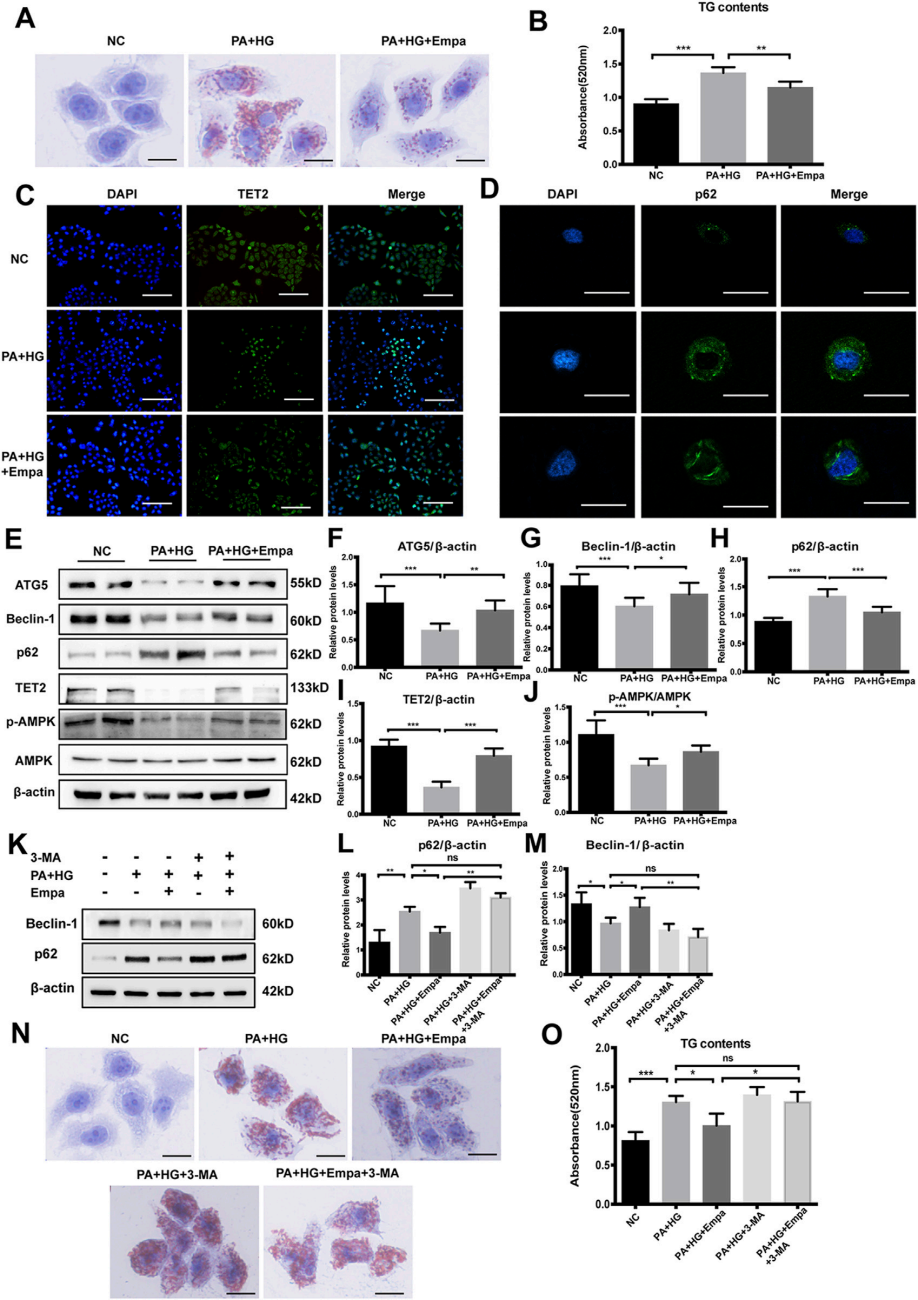
Empagliflozin ameliorates lipid accumulation and activates autophagy and the AMPK-TET2 signaling in HL7702 cells treated with PA and HG **(A–J)** HL7702 cells were treated with or without PA and HG, with or without empagliflozin **(A)** Representative images of Oil Red O staining and **(B)** quantitative analysis of TGs. Scale bar represents 30 μm. Immunofluorescence of **(C)** TET2 and **(D)** p62 in HL7702 cells. Scale bars represent 30 μm **(C)** and 100 μm **(D)**, respectively, **(E)** Western blot analyses of ATG5, Beclin-1, p62, TET2, p-AMPK, and AMPK with β-actin as a loading control and **(F–J)** densitometric analyses of band intensities normalized to β-actin **(K–O)** HL7702 cells were treated with or without PA and HG, with or without empagliflozin, in the presence or absence of 3-MA **(K)** Western blot analyses of Beclin-1 and p62 with β-actin as a loading control and **(L, M)** densitometric analyses of band intensities normalized to β-actin **(N)** Representative images of Oil Red O staining and **(O)** quantitative analyses of TGs. Data in **(B, F–J, L, M**, and **O)** are presented as means ± SEM from three independent experiments. Empa, empagliflozin; HG, high glucose; NC, negative control group; PA, palmitic acid; TG, triglyceride; 3-MA, 3-methyladenine. *p < 0.05, **p < 0.01, ***p < 0.001.

In the published article, there was an error in Figure 4 as published. The authors incorrectly used the image of the PA + HG + Empa group while preparing the image of the PA + HG + AICAR group (Figure 4A). The corrected Figure 4 and its caption appear below.

**FIGURE 4:**
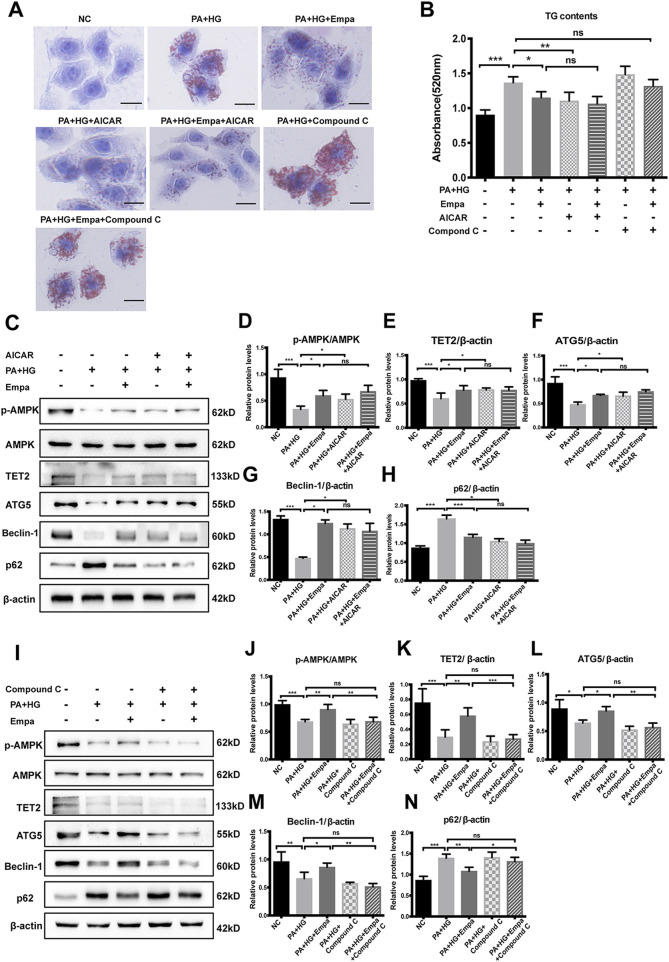
The increase in TET2 and autophagy and reduction in lipid accumulation by empagliflozin are dependent on AMPK activation. HL7702 cells were treated with or without PA and HG, with or without empagliflozin, in the presence or absence of AICAR or compound C **(A)** Representative images of oil red O staining and **(B)** quantitative analysis of TGs. Scale bar represents 30 μm **(C, I)** Western blot analyses of p-AMPK, AMPK, TET2, ATG5, Beclin-1, and p62 with β-actin as a loading control and **(D–H, J–N)** densitometric analyses of band intensities normalized to β-actin. Data in **(B, D–H, J–N)** are presented as means ± SEM from three independent experiments. Empa, empagliflozin; HG, high glucose; NC, negative control group; ns, not significant; PA, palmitic acid; TG, triglyceride. *p < 0.05, **p < 0.01, ***p < 0.001.

In the published article, there was an error in **Supplementary Data Sheet 1**. An error was found in the western blot of the β-actin protein panel in **Supplementary Figure 5A**. The corrected **Supplementary Figure 5** can be found in the Supplementary Material link of the original article.

The authors apologize for these errors and state that this does not change the scientific conclusions of the article in any way. The original article has been updated.

