# Supplementary material for: Empagliflozin Alleviates Hepatic Steatosis by Activating the AMPK-TET2-Autophagy Pathway in vivo and in vitro
Source: Front Pharmacol. 2021 Jan 20;11:622153. doi: 10.3389/fphar.2020.622153 (PMC7854384; doi:10.3389/fphar.2020.622153)
Supplement: Supplementary file 1 [file DataSheet2.docx]

**Supplemental 2**

**Supplementary Figure 7**

**A**

**
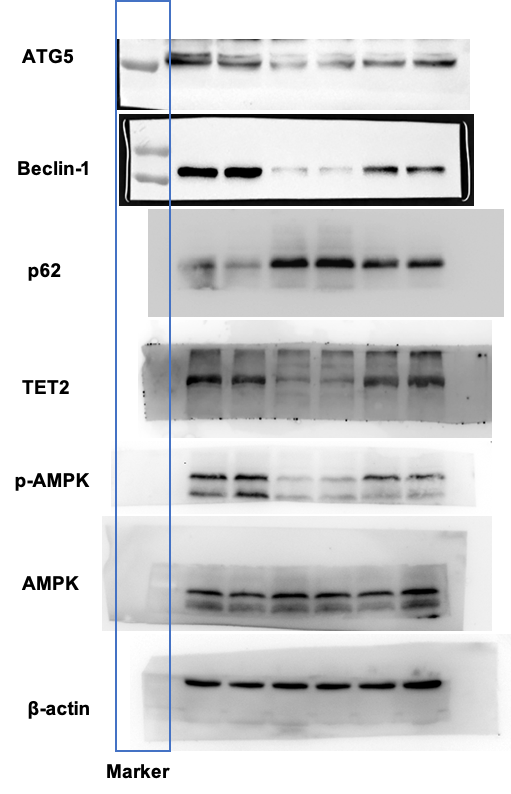
**

**Supplementary Figure 7** The underlying data of Figure 2 is above.

**Supplementary Figure 8**

**A**

**
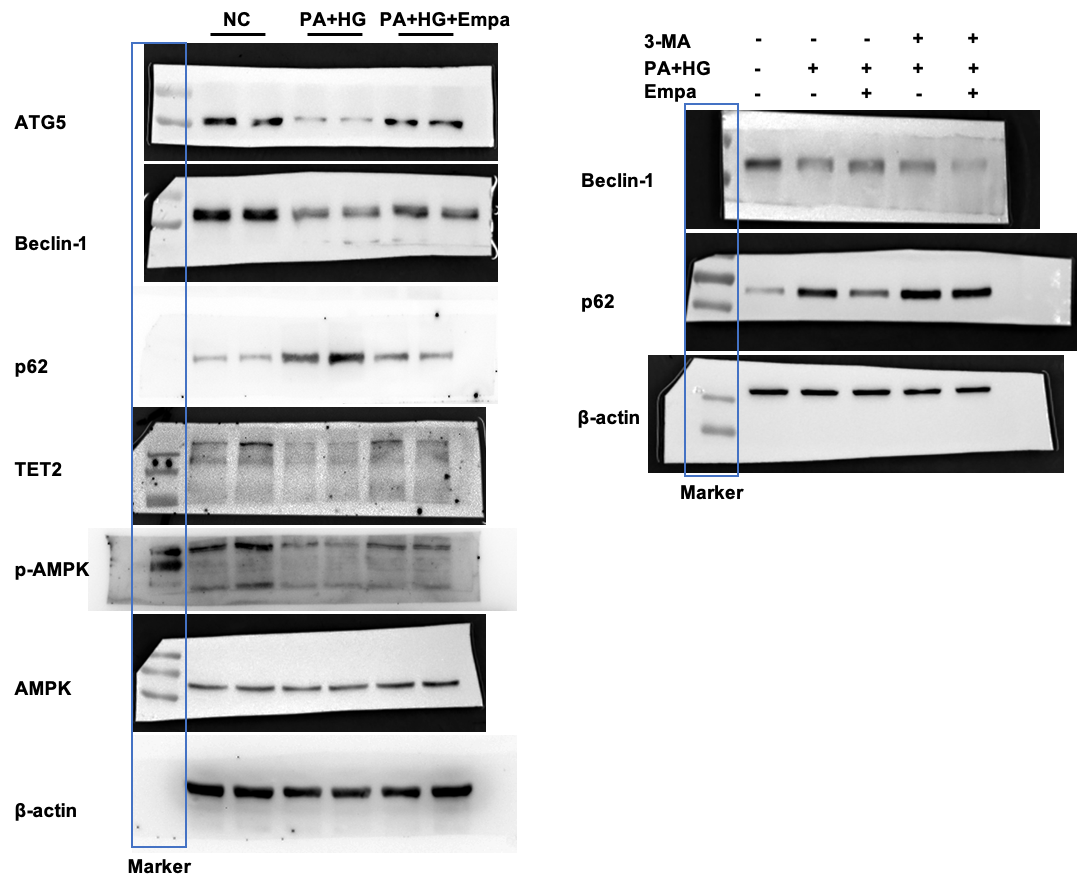
**

**Supplementary Figure 8** The underlying data of Figure 3 is above.

**Supplementary Figure 9**

**A**


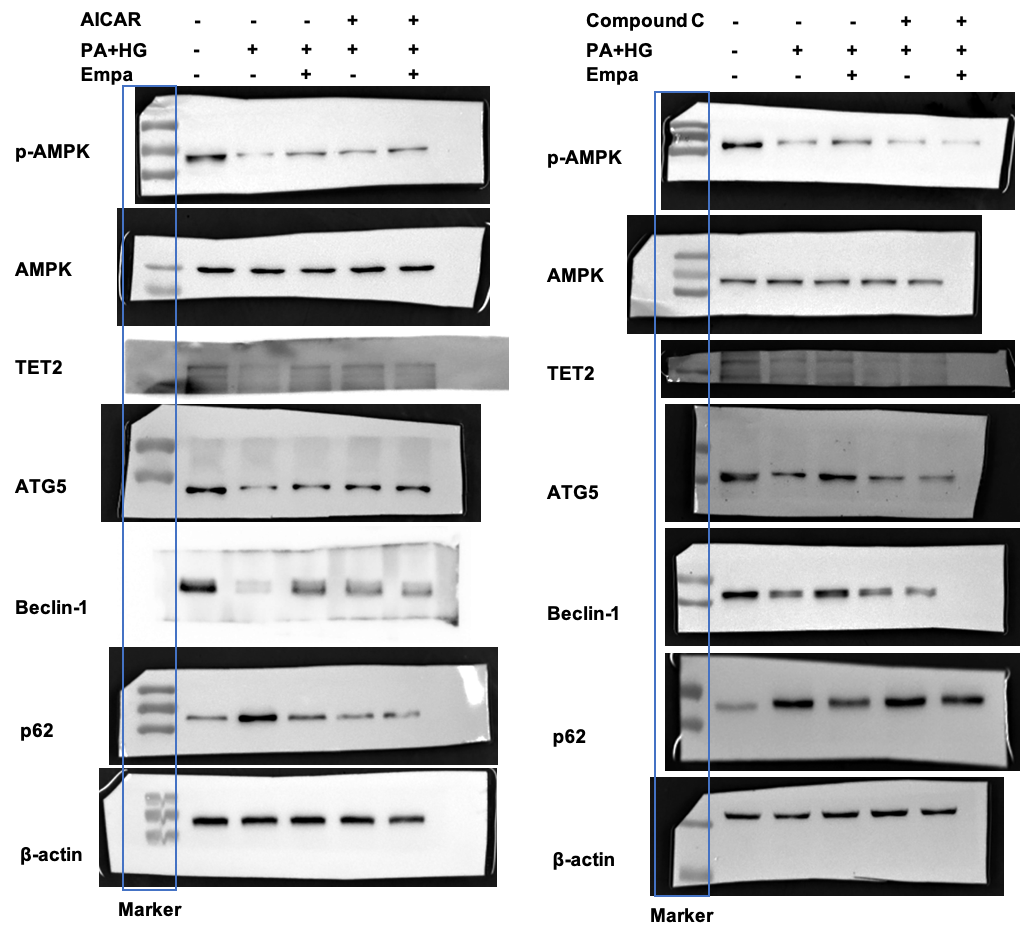


**Supplementary Figure 9** The underlying data of Figure 4 is above.

**Supplementary Figure 10**

**A**


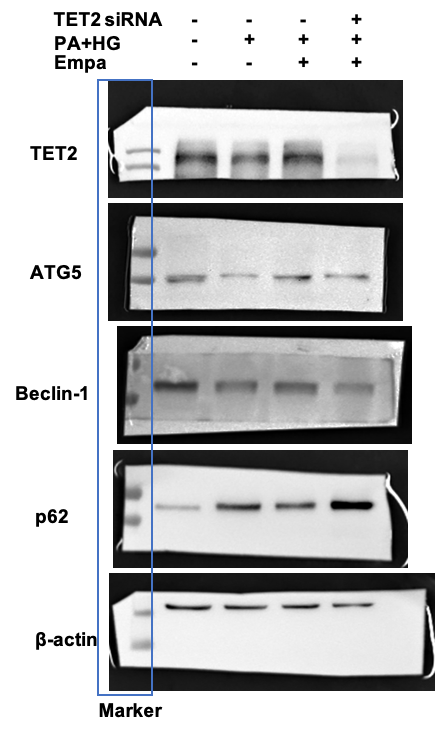


**Supplementary Figure 10** The underlying data of Figure 5 is above.
