# Supplementary material for: Empagliflozin Alleviates Hepatic Steatosis by Activating the AMPK-TET2-Autophagy Pathway in vivo and in vitro
Source: Front Pharmacol. 2021 Jan 20;11:622153. doi: 10.3389/fphar.2020.622153 (PMC7854384; doi:10.3389/fphar.2020.622153)
Supplement: Supplementary file 2 [file DataSheet1.docx]

**Supplemental Figure 1**

**
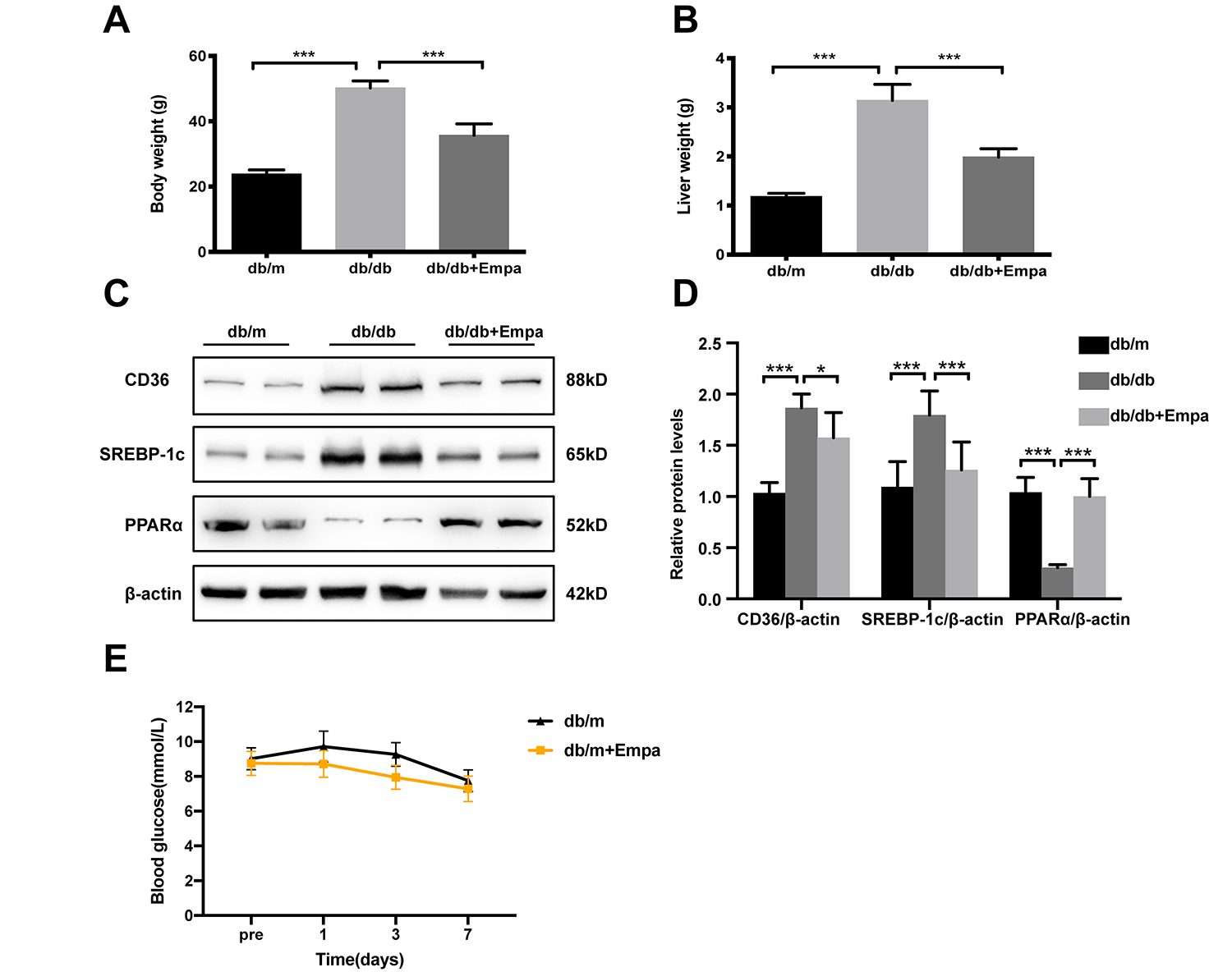
**

**Supplementary Figure 1.** Empagliflozin decreases body weight, liver weight and lipid accumulation in db/db mice. Eight-week-old male db/db mice were treated with saline or empagliflozin for eight weeks, and eight-week-old male db/m mice given no treatment or short-term (7 days) empagliflozin were used as a control. (A) Body weight and (B) liver weight were measured after 8-week treatment. (C) Western blot analyses of CD36, SREBP-1c and PPARα with β-actin as a loading control and (D) densitometric analyses of band intensities normalised to β-actin. (E) Blood glucose concentrations over the 7-day treatment duration. Data are in (A, B, D, E) presented as means ± SEM. Empa, empagliflozin. * *p* < 0∙05, *** *p* < 0∙001**.**

**Supplemental Figure 2**

**
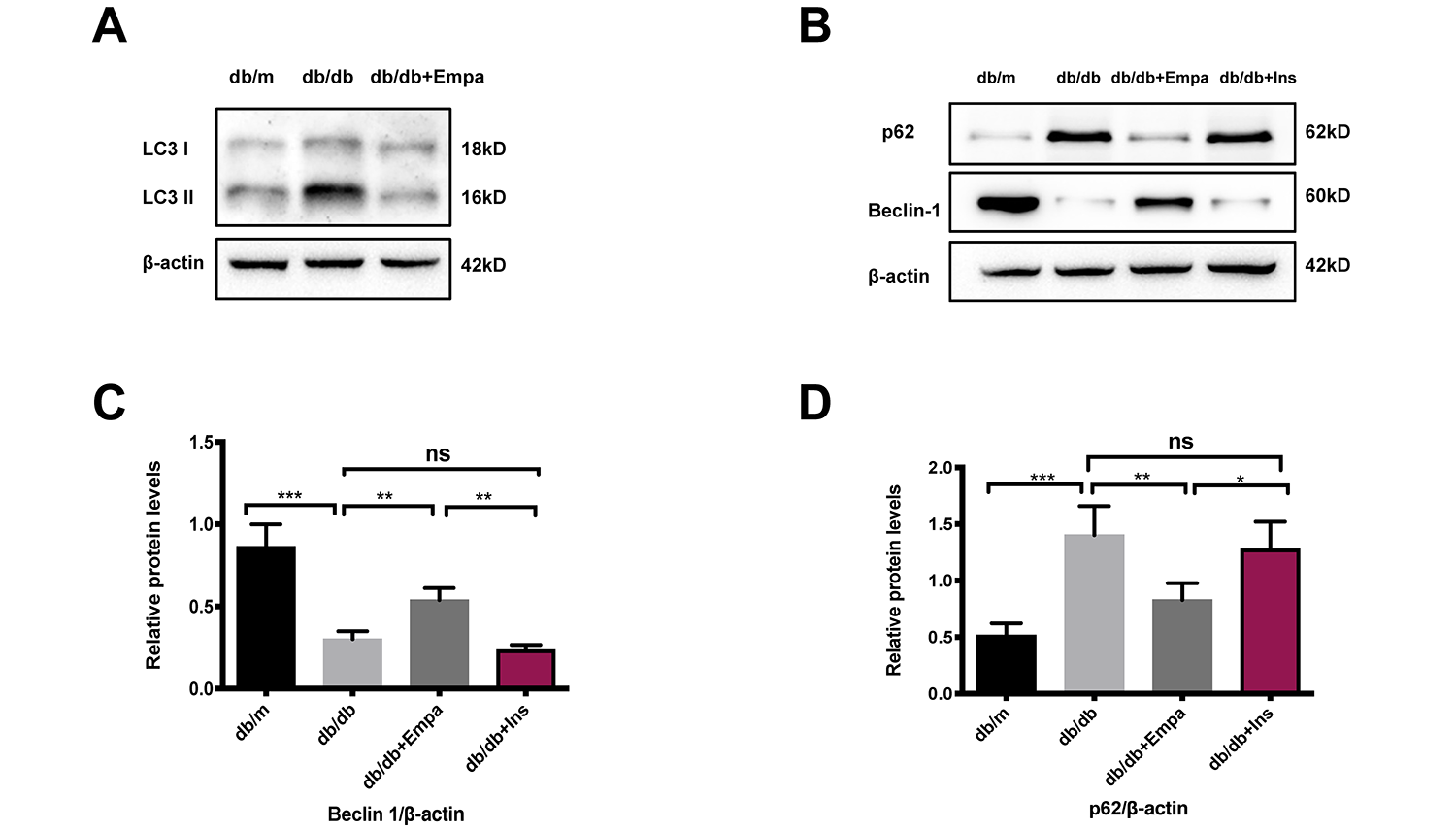
**

**Supplementary Figure 2.** Empagliflozin but not insulin restores autophagy activity in db/db mice. Eight-week-old male db/db mice were treated with saline, empagliflozin, or insulin for eight weeks, and eight-week-old male db/m mice given no treatment were used as a control. Western blot analyses of (A) LC3 and (B) p62 and Beclin-1 with β-actin as a loading control. (C, D) Densitometric analyses of band intensities normalised to β-actin. Data in (C) and (D) are presented as means ± SEM from three independent experiments. Empa, empagliflozin; Ins, insulin glargine; ns, not significant. * *p* < 0∙05, ** *p* < 0∙01, *** *p* < 0∙001**.**

**Supplemental Figure 3**

**
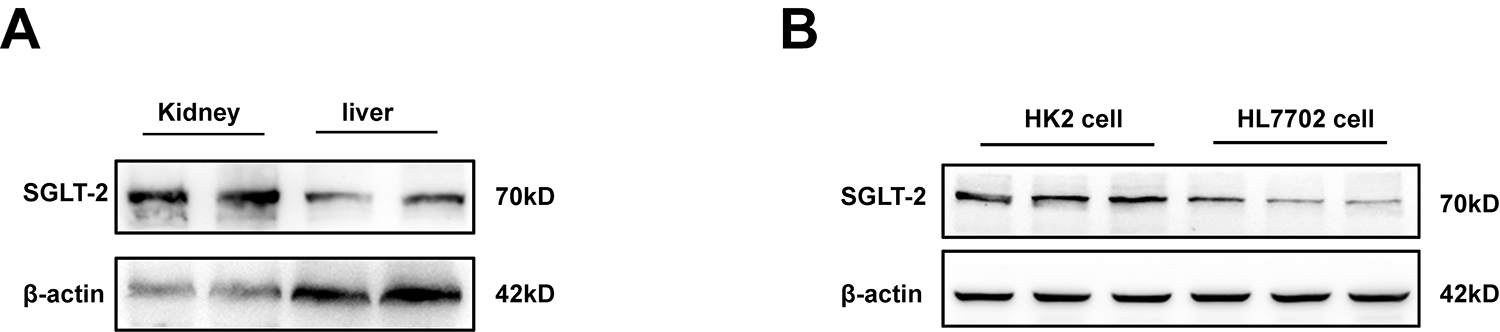
**

**Supplementary Figure 3.** SGLT-2 is expressed in both mice livers and HL7702 cells. (A, B) Western blot analyses of SGLT-2 with β-actin as a loading control.

**Supplementary Figure 4**


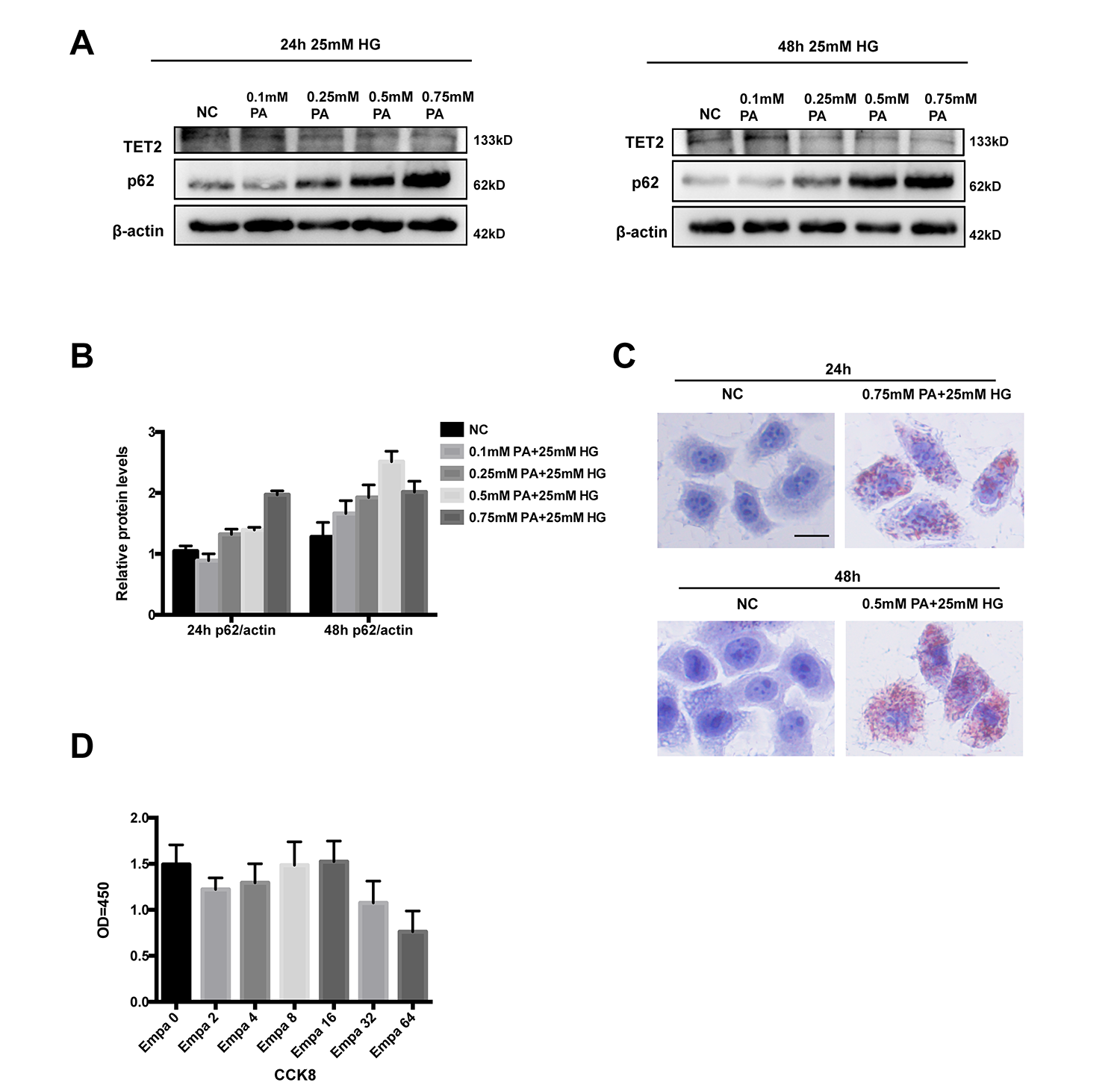


**Supplementary Figure 4.** PA and HG induce steatosis in HL7702 cells. (A–C) HL7702 cells were treated with different concentrations of PA and 25 mM glucose for different durations. (A) Western blot analyses of TET2 and p62 with β-actin as a loading control and (B) densitometric analyses of band intensities normalised to β-actin. (C) Representative images of Oil Red O staining. Scale bar represents 30 μm. (D) Cell viability measured by CCK8 after being treated with different doses of empagliflozin. Data in (B and D) are presented as means ± SEM from three independent experiments. Empa, empagliflozin; Glu, glucose; HG, high glucose; NC, negative control group; PA, palmitic acid.

**Supplementary Figure 5**


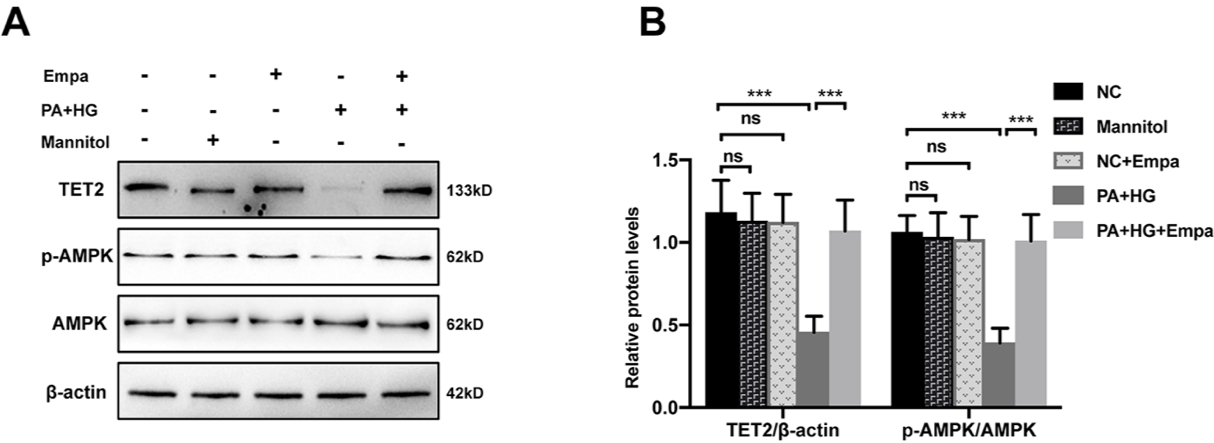


**Supplementary Figure 5.** Empagliflozin ameliorates lipid accumulation and activates autophagy and the AMPK-TET2 signaling in HL7702 cells treated with PA and HG. HL7702 cells were treated with or without mannitol, with or without PA and HG, with or without empagliflozin. (A) Western blot analyses of p-AMPK, AMPK and TET2 with β-actin as a loading control and (B) densitometric analyses of band intensities normalised to β-actin. Data in (B) are presented as means ± SEM from three independent experiments. Empa, empagliflozin; HG, high glucose; NC, negative control group; PA, palmitic acid.

**Supplementary Figure 6**


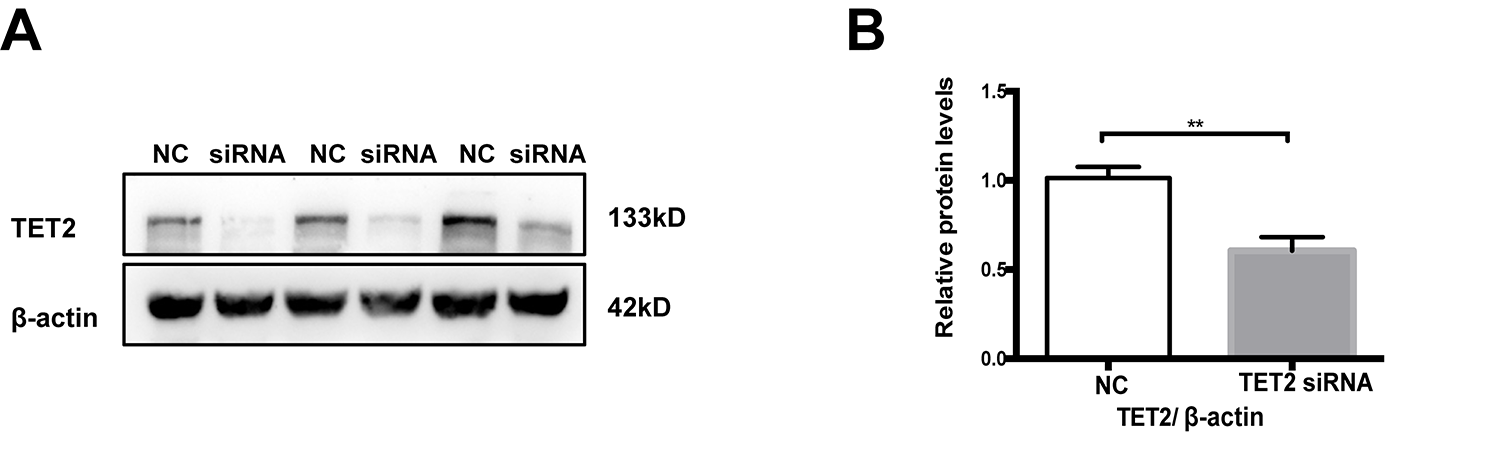


**Supplementary Figure 6.** Knockdown of TET2 by TET2 siRNA transfection in HL7702 cells. (A) Western blot analysis of TET2 with β-actin as a loading control and (B) densitometric analyses of band intensities normalised to β-actin. Data in (B) are presented as means ± SEM from three independent experiments. ** *p* < 0∙01.
